# Metabolomics analysis elucidates unique influences on purine / pyrimidine metabolism by xanthine oxidoreductase inhibitors in a rat model of renal ischemia-reperfusion injury

**DOI:** 10.1186/s10020-019-0109-y

**Published:** 2019-08-22

**Authors:** Takashi Tani, Ken Okamoto, Megumi Fujiwara, Akira Katayama, Shuichi Tsuruoka

**Affiliations:** 1 Department of Nephrology, Graduate School of Medicine, Nippon Medical School, 1-1-5 Sendagi, Bunkyo-ku, Tokyo, 113-8602 Japan; 2 Department of Metabolism and Nutrition, Graduate School of Medicine, Nippon Medical School, 1-1-5 Sendagi, Bunkyo-ku, Tokyo, 113-8602 Japan

**Keywords:** Metabolome, Xanthine oxidoreductase inhibitor, Ischemia-reperfusion injury

## Abstract

**Background:**

Clinically applied as anti-gout drugs, xanthine oxidoreductase (XOR) inhibitors, especially the potent, selective, non-purine-analog XOR inhibitors febuxostat and topiroxostat, exert organ-protective effects. We tested the hypothesis that preservation of tissue concentrations of high-energy phosphates, such as ATP and ADP, contributes to organ-protective effects through CE-TOFMS metabolomics.

**Methods:**

Rats were subjected to 30 min of renal ischemia-reperfusion (I/R) injury 60 min after oral administration of 10 mg/kg febuxostat, 10 mg/kg topiroxostat, 50 mg/kg allopurinol, or vehicle.

**Results:**

In non-purine-analog XOR inhibitor-treated groups, renal concentrations of high-energy phosphates were greater before and after I/R injury, and renal adenine compounds were less depleted by I/R injury than in the vehicle and allopurinol groups. These findings were well in accordance with the proposed hypothesis that the recomposition of high-energy phosphates is promoted by non-purine-analog XOR inhibitors via the salvage pathway through blockade of hypoxanthine catabolism, whereas non-specific inhibitory effects of allopurinol on purine/pyrimidine enzymes impede this re-synthesis process.

**Conclusions:**

This metabolic approach shed light on the physiology of the organ-protective effects of XOR inhibitors.

**Electronic supplementary material:**

The online version of this article (10.1186/s10020-019-0109-y) contains supplementary material, which is available to authorized users.

## Background

Xanthine oxidoreductase (XOR) catalyzes the oxidation of hypoxanthine to xanthine and that of xanthine to uric acid, as well as the reduction of nicotinamide adenine dinucleotide (NAD^+^) or molecular oxygen. Because they inhibit the conversion of xanthine to uric acid, XOR inhibitors are used as anti-gout drugs. Clinically used XOR inhibitors are classified into two groups based on different chemical structures and inhibition mechanisms: the purine-analog inhibitor allopurinol, and non-purine-analog inhibitors, such as febuxostat and topiroxostat. The largest difference between the purine analog and non-purine-analog inhibitors is the specificity of the target enzyme; the non-purine-analog inhibitors impede the activity of XOR solely by obstructing substrate binding, and do not inhibit additional enzymes in purine and pyrimidine metabolism pathways as reported for allopurinol (Okamoto et al. 2003; Takano et al. 2005).

XOR inhibitors have shown potential organ-protective effects in clinical trials (Sezai et al. 2015; Tanaka et al. 2015; Tsuruta et al. 2014; Whelton et al. 2011) as well as animal experiments (Sanchez-Lozada et al. 2008; Tsuda et al. 2012; Omori et al. 2012). The mechanism of protection is usually explained by the oxidative-stress hypothesis: potent inhibition of XOR activity results in suppression of the activity of xanthine oxidase (XO), where XO functions in disease states by transferring electrons to O_2_ to form O_2_^−^, leading to oxidative stress and resulting in organ disorders (Tsuda et al. 2012; Omori et al. 2012; McCord 1985).

There are several clinical reports indicating that the non-purine-analog inhibitor, febuxostat shows superior organ-protective effects compared to allopurinol (Sezai et al. 2015; Kim et al. 2017; Chou et al. 2017; Foody et al. 2017; Shafik 2013; Wang et al. 2015; Khan et al. 2017; Kato et al. 2016). Some in vivo experiments revealed a superior organ-protective effect of febuxostat compared to allopurinol in an intestinal ischemia-reperfusion (I/R) injury rat model, a myocardial I/R injury mouse/rat model and a mouse model of amyotrophic lateral sclerosis (ALS) (Shafik 2013; Wang et al. 2015; Khan et al. 2017; Kato et al. 2016). Most of these reports claim that superiority of non-purine-analog inhibitor to allopurinol was due to significant potency of non-purine-analog inhibitor to inhibit XOR activity, and thus lowering oxidative-stress. However, there are no studies in which augmentation of XO activity itself was confirmed enzymatically, and the specific mechanism of action remains further to be elucidated.

One possible alternate answer to the question is that XOR inhibitors might exhibit organ-protective effects by affecting purine metabolism. Indeed, administration of allopurinol maintained tissue concentrations of ATP, adenosine diphosphate (ADP), and adenosine 5′-monophosphate (AMP), and preserved functional organ activity (Cunningham et al. 1974; Lasley et al. 1988; Khatib et al. 2001). As XOR is a key player in purine metabolism, exhaustive metabolic analysis would contribute to elucidate the beneficial organ-protective effect of XOR inhibitors. To survey such global alterations of metabolic pathways, metabolomics is considered an appropriate approach. The metabolome is the global collection of small molecules (typically < 1500 Da; e.g., sugars, amino acids, organic acids, nucleotides, acylcarnitines, and lipids) in a cell or a biologic specimen (Kalim and Rhee 2017).

Animal models of I/R injury have long been used to evaluate metabolic fluctuations in organ disorders (Cunningham et al. 1974; Lasley et al. 1988; Khatib et al. 2001; Stromski et al. 1986; Stromski et al. 1988; Okabe 1996). By investigating the influence of renal I/R on the metabolome, I/R-induced metabolic changes can be elucidated. For instance, ischemia produces a rapid loss of high-energy phosphates and accumulation of hydrolysis products, including lactate, β-hydroxybutyrate, and citrate (Weiner 1987).

The influence of XOR inhibitors on metabolic pathways may be related to their organ-protective effects (Cunningham et al. 1974; Lasley et al. 1988; Khatib et al. 2001); thus, clarifying the alterations in the metabolome may help elucidate their mechanism of action. Therefore, in this study, we aimed to elucidate the mechanism of XOR inhibitors using capillary electrophoresis–time-of-flight mass spectrometry (CE-TOFMS) in a rat model of renal I/R, in which the effectiveness of XOR inhibitors has been established (Tsuda et al. 2012).

## Methods

### Rat model of renal I/R injury

Sixty-two 6-week-old male Sprague–Dawley rats were randomly allocated to four groups (Table 1): (1) vehicle-treatment group (Veh; *n* = 17); (2) febuxostat-treatment group (Feb; *n* = 15); (3) topiroxostat-treatment group (Top; n = 15) (4) allopurinol-treatment group (Allo; n = 15). Vehicle-treated rats orally received 0.5 ml of 0.5% methylcellulose 60 min before surgery. Experimental rats were orally administered 10 mg/kg febuxostat, 10 mg/kg topiroxostat, or 50 mg/kg allopurinol in 0.5% methylcellulose, 60 min before operation.

**Table 1:** Overview of the experimental set-up and plasma biochemical data

	Veh-S	Feb-S	Top-S	Allo-S	Veh-I	Feb-I	Top-I	Allo-I	Veh-R	Feb-R	Top-R	Allo-R
Sampling state	Stationary (under resting state)				30 min left renal ischemia				30 min left renal ischemia + 30 min reperfusion			
Administrated drugs	0.5% Methyl-cellulose	10 mg/kg Febu-xostat	10 mg/kg Topiro-xostat	50 mg/kg Allo-purinol	0.5% Methyl-cellulose	10 mg/kg Febu-xostat	10 mg/kg Topiro-xostat	50 mg/kg Allo-purinol	0.5% Methyl-cellulose	10 mg/kg Febu-xostat	10 mg/kg Topiro-xostat	50 mg/kg Allo-purinol
Identification of group												
BUN (mg/dl)	16.8 ± 1.6	12.8 ± 1.0	14.7 ± 2.5	16.1 ± 1.4	21.4 ± 2.9 ^$$,&&,##^	20.2 ± 2.1 ^$$,&&,#^	20.1 ± 3.0 ^$$,&&,##^	21.9 ± 3.2 ^*,$$,&&,##^	20.2 ± 2.0 ^$$,&&,#^	20.3 ± 0.7 ^$$,&&,#^	20.5 ± 2.9 ^$$,&&,#^	21.2 ± 1.7 ^*,$$,&&,##^
Creatinine (mg/dl)	0.26 ± 0.03	0.28 ± 0.03	0.30 ± 0.03	0.28 ± 0.04	0.41 ± 0.04 **^,$$,&,##^	0.39 ± 0.05 **^,$$,&,##^	0.41 ± 0.05 **^,$$,&&,##^	0.43 ± 0.09 **^,$$,&&,##^	0.39 ± 0.05 **^,$$,&,##^	0.37 ± 0.05 **^,$,##^	0.45 ± 0.04 **^,$$,&&,##^	0.43 ± 0.06 **^,$$,&&,##^
Uric Acid (mg/dl)	0.46 ± 0.17	0.1 > ±N.A	0.1 > ±N.A	0.1 > ±N.A	0.63 ± 0.14*	0.1	0.1 > ±N.A	0.1 > ±N.A	0.54 ± 0.05	0.1	0.1 > ±N.A	0.1

*BUN*, blood urea nitrogen. Concentrations are expressed as nmol/g wet weight; the data are represented as the mean ± SEM (n = 5/6). *P < 0.05 and **P < 0.01 versus Veh-S; ^$^*P* < 0.05 and ^$$^*P* < 0.01 versus Feb-S; ^&^*P* < 0.05 and ^&&^*P* < 0.01 versus Top-S; ^#^*P* < 0.05 and ^##^*P* < 0.01 versus Allo-S; one-way ANOVA followed by Games–Howell’s test

Before surgery, rats were anesthetized by intraperitoneal injection of 360 mg/kg chloral hydrate (Wako Pure Chemical Industries, CAS: 302–17-0) and were placed on a warmed table to maintain a rectal temperature of 37 °C. The animals were then allowed to stabilize for 20 min. I/R injury was initiated by left renal pedicle occlusion with a non-traumatic vascular clamp for 30 min, during which time the kidney was kept warm and moist. Occlusion was confirmed visually by a color change to a paler shade. Then, the clamp was removed, and the kidney was observed for return of blood flow.

Kidney samples were obtained at different phases of the operation (Table 1): (a) stationary phase (*n* = 21), sacrifice at 60 min after drug administration with no ischemic damage (S groups); (b) ischemic phase (*n* = 20), sacrifice at after 30 min left renal ischemia (I groups); (c) reperfusion phase (n = 21), sacrifice after 30 min of left renal ischemia followed by 30 min of reperfusion (R groups). Each sample group (*n* = 5 or 6) was named after the administered drug and sampling phase, e.g., Feb-I represents the group treated with febuxostat that underwent 30 min of I/R injury. At the time of sacrifice, the left kidney was quickly weighed, snap-frozen in liquid nitrogen, and stored at − 80 °C, and blood was obtained via puncture of the inferior vena cava. Plasma creatinine and urea were measured enzymatically and by the urease-GLDH method, respectively, on a Hitachi 7180 auto-analyzer (Hitachi High-Technologies, Tokyo, Japan). Rats were euthanized through cervical dislocation under anesthesia.

### Preparation of kidney extracts for HPLC and metabolome analyses

Special care was taken throughout the procedure not to denature adenine nucleotides. Frozen samples were crushed into powder using a Cryopress (CP-100w; Microtec, Chiba, Japan). The samples were transferred into ice-cold 70% acetonitrile (1:5, v/v), vortexed immediately for 30 s, and centrifuged (10 min, 4 °C, 20,670×*g*). The supernatant was stored at − 80 °C.

### Measurement of purine nucleotide concentration by HPLC

Purine nucleotide concentration of kidney extract (10 μl) was measured using a high-performance liquid chromatography (HPLC) system (ÄKTApurifier UPC 10; GE Healthcare UK/Amersham, Little Chalfont, Buckinghamshire, UK) with a reverse-phase column (Supelcosil LC-18-T, 250 × 4.6 mm, 5 μm; Sigma-Aldrich, Bellefonte, PA, USA) and a guard column (Supelguard LC-18-T, 20 × 4.0 mm; Sigma-Aldrich). All concentrations are expressed as nmol/g wet weight. For details of the measurement, see supporting information.

### Measurement of metabolites

CE-TOFMS was performed on an Agilent CE Capillary Electrophoresis System equipped with an Agilent 6210 TOF mass spectrometer, Agilent 1100 isocratic HPLC pump, Agilent G1603A CE-MS adapter kit, and Agilent G1607A CE-ESI-MS sprayer kit (Agilent Technologies, Waldbronn, Germany). Metabolome measurements were performed at Human Metabolome Technologies as previously described (Soga and Heiger 2000; Soga et al. 2002; Soga et al. 2003). For details of the measurement, see supporting information.

### Absolute quantification of metabolites and statistical analyses

Absolute quantification was performed for 110 metabolites, including glycolytic and TCA cycle intermediates, amino acids, and nucleic acids, as previously described (Subramanian et al. 2017). All metabolite concentrations were calculated by normalizing the peak area of each metabolite to the area of the internal standard and by using standard curves obtained by single-point (100 μM) calibrations. Hierarchical cluster analysis, principal component analysis (PCA), and PLS-ROG analysis were performed using proprietary software, PeakStat and SampleStat, respectively. Partial least squares with rank order of groups (PLS-ROG) is an extended version of PLS that adds a differential penalty between means of groups in the PLS subspace, which can distinguish between groups and can reflect group rank order (Yamamoto n.d.). Detected metabolites were plotted on metabolic pathway maps using Visualization and Analysis of Networks containing Experimental Data software (Junker et al. 2006). Based on the concentration of each metabolite measured by CE-TOFMS, energy charge, total adenine nucleotide (TAN) and TAN' were calculated by the following formulas:

$$ {}_{\mathrm{Energy}\ \mathrm{charge}=}\frac{\mathrm{ATP}+0.5\mathrm{ADP}}{\mathrm{ATP}+\mathrm{ADP}+\mathrm{AMP}} $$

TAN = ATP + ADP + AMP, TAN' = dATP + phosphoribosyl diphosphate (PRPP) + adenosine + adenine + inosine + IMP + hypoxanthine + xanthine + uric acid. ΔhTAN + TAN' was calculated as the TAN + TAN' of the reperfused state minus that of the ischemic state.

### Western blotting and XOR activity assay of kidney

For western blotting of kidney lysates, we followed the method used in our previous reports, with minor modifications (Okabe 1996; Ikegami and Nishino 1986). For details of the method, see supporting information. The XOR activity of tissue lysates was measured spectrophotometrically by following the increase in the absorbance of uric acid at 295 nm (Okabe 1996; Ikegami and Nishino 1986). The assay buffer consisted of 50 mM potassium phosphate buffer (pH 7.8), 0.4 mM EDTA, 0.15 mM xanthine, 0.5 mM NAD^+^, and 1 mM oxonic acid. All measurements were performed at 25 °C under air-saturated conditions.

### Quantitative reverse-transcription (qRT-)PCR

RNA was extracted from frozen tissue samples, and complementary DNA was generated with oligo-dT primers using an RNeasy Mini Kit (Qiagen GmbH, Hilden, Germany) and a ReverTra qPCR RT Kit (Toyobo Co., Osaka, Japan) according to the manufacturer’s protocol, respectively. qPCR amplification was performed using the TaqMan Fast Advanced Master Mix (Life Technologies, Carlsbad, CA, USA) in 96-well optical plates on an ABI 7500 Fast Real-Time PCR System (Life Technologies). Gene expression was normalized to levels of 18S rRNA as an internal control and expressed as fold increases using the ΔΔCt method. For details of the method, see supporting information.

### Statistics

Statistical analyses were performed using SPSS version 16.0 (IBM, Chicago, IL, USA), with values expressed as the mean ± standard error of the mean (SEM), unless otherwise stated. For analysis of biochemical data, HPLC and qRT-PCR results, one-way analysis of variance (ANOVA) followed by Games-Howell’s test was used to find statistical differences among groups, and *p* < 0.05 was considered significant. For an untargeted approach in the metabolome analysis, one-way ANOVA was applied to identify metabolites showing statistical differences across groups. The *q*-value, determined by using the Benjamini–Hochberg correction, was applied to adjust for false positive discovery arising from multiple testing of *p*-values (adjusted for predicted *p* < 0.05) (Benjamini and Hochberg 1995; Storey 2003).

### Study approval

All experiments using rats were conducted in compliance with the guidelines for animal experiments of Nippon Medical School, and the study protocol was approved by the Institutional Animal Care and Use Committee at Nippon Medical School (approval number 27–004).

## Results

### XOR activity and western blotting analysis of kidney extracts during I/R injury

**Fig. 1:**
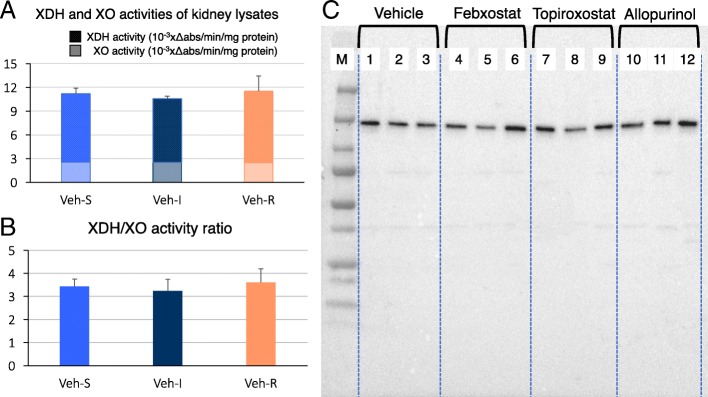
XOR activity assays and XOR expression in kidney lysates. XOR activity was not detected in kidney lysates of all XOR inhibitor-treated groups. **a** XDH and XO activity in vehicle-treated rats did not change during I/R injury. **b** XDH/XO ratios were not altered throughout I/R. **c** XOR expression in kidney lysates was detected as a 150-kDa single band in each group by western blotting. M, molecular markers; 1–3, vehicle-treated rats; 4–6, febuxostat-treated rats; 7–9, topiroxostat-treated rats; 10–12, allopurinol-treated rats. Samples in lanes 1, 4, 7, and 10 are kidney samples in the stationary state; those in lanes 2, 5, 8, 11 are at 30 min ischemia injury; and those in lanes 3, 6, 9, 12 are at 30 min of reperfusion after 30 min of ischemia injury

Western blotting for XOR expression in kidney lysates showed a single 150-kDa band in each group, and the amount enzyme protein was not changed throughout the I/R process, this indicates that no limited proteolysis of XOR nor enzyme induction occurred (Fig. 1c). XOR activities were not changed in the kidney extracts of all I/R stages of vehicle-treated rats (Fig. 1a). The xanthine dehydrogenase (XDH)/xanthine oxidase (XO) activity ratio and total XOR (XDH + XO), XDH activities from vehicle-treated rats showed no significant changes during I/R (Fig. 1a, b). No XOR activities were detected in all the XOR inhibitor-treated rats.

### mRNA expression levels of key enzymes in purine metabolism and glycolysis/gluconeogenesis during I/R injury

We evaluated the mRNA expressions of key enzymes in the stationary and reperfused phases (Additional file 1: Figure S1). No significant differences in the expression of adenine phosphoribosyltransferase (*APRT*), *XOR*, pyruvate kinase (*PK*), phosphofructokinase (*PFK*), pyruvate dehydrogenase kinase (*PDH*), pyruvate carboxylase (*PC*), and phosphoenolpyruvate carboxykinase (*PEPCK*) within groups in either the stationary or reperfused state were observed (Additional file 1: Figure S1A, C–H). Hypoxanthine-guanine phosphoribosyltransferase (*HGPRTase*) expression decreased in the Top-R group as compared to that in the Feb-R and Allo-R groups (Additional file 1: Figure S1B).

### HPLC analysis of purine metabolites

**Fig. 2:**
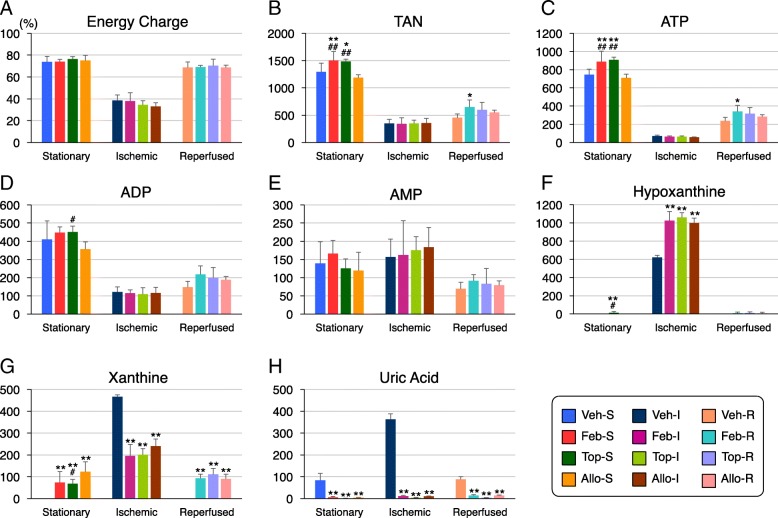
Purine nucleotide concentrations measured by HPLC. Concentrations of ATP, ADP, AMP, hypoxanthine, xanthine, and uric acid were measured by HPLC. In the stationary state, ATP and TAN levels were significantly higher in the Feb-S and Top-S groups than in the Veh-S and Allo-S groups (**b**, **c**), whereas xanthine was not detected (ND) in the Veh-S group, and uric acid levels decreased in the inhibitor-treated groups (**g**, **h**). ADP levels increased in the Feb-S and Top-S group compared with that in the Veh-S group (**d**). In the ischemic state, energy charge and concentrations of purine nucleotide decreased considerably from the stationary condition (**a**, **b**, **c**, **d**); hypoxanthine levels were higher, and xanthine and uric acid levels were lower in the inhibitor-treated groups (**f**, **g**, **h**). In the reperfused state, TAN and ATP levels were higher in the Feb-R group than in the Veh-R group (B, C); hypoxanthine and xanthine remained positive only in inhibitor-treated groups (**f**, **g**), and uric acid in Veh-R rats returned to the stationary level (**h**). Energy charge and TAN were calculated as follows: energy charge = ATP + 0.5*ADP/ATP + ADP + AMP, TAN = ATP + ADP + AMP. Concentrations are expressed as nmol/g wet weight; the data are presented as the mean ± SEM (*n* = 5~6). *P < 0.05 and **P < 0.01 versus Veh; #P < 0.05 and ##P < 0.01 versus Allo; one-way ANOVA followed by Games–Howell’s test

Concentrations of ATP, ADP, AMP, hypoxanthine, xanthine, and uric acid in kidney extracts were measured by HPLC method (Fig. 2; for the specific concentration of each metabolite, see Additional file 1: Table S1). In the resting phase, ATP concentrations of Feb-S and Top-S are significantly higher than Veh-S (19.4 and 22.0%, respectively). ADP concentrations are also 9.3 and 10.0% higher in Feb-S and Top-S than Veh-S. On the other hand, no such alternation was observed in the Allo-S group. Urate concentrations of inhibitor-treated groups are all significantly lower than Veh-S. In the ischemic phase, ATP and ADP were drastically reduced and concomitant increase of hypoxanthine and xanthine were observed (Fig. 2c, d, f, g). Hypoxanthine levels were higher, and xanthine and uric acid levels were lower in the inhibitor-treated groups (Fig. 2f-h). TAN and energy charge were comparably decreased in all groups (Fig. 2a, b), and the energy charge was decreased to 33.0–38.5% (Fig. 2a). In the reperfused state, energy charge, TAN, ATP, and ADP were recovered from the ischemic state, but not high enough to the stationary state levels, with recovery rates of 91.4–93.4%, 35.2–46.5%, 32.0–40.0%, and 36.1–48.7%, respectively (Fig. 2a–e); the recovery rates of all the detected purine metabolites were the lowest in the vehicle group (Veh-R).

### CE-TOFMS metabolomics reveals alternations on purine metabolites by XOR inhibitors

**Fig. 3:**
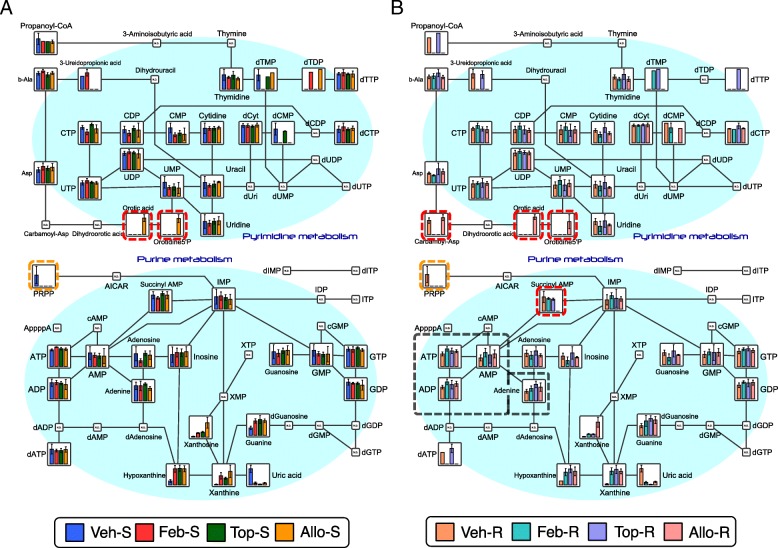
Purine/pyrimidine metabolism pathway map in stationary and reperfused states. Pathway maps of purine/pyrimidine metabolism in both stationary (**a**) and reperfused (**b**) states show alterations in the concentrations of several metabolites. Uric acid in XOR inhibitor-treated groups was maintained at very low concentrations, whereas hypoxanthine and xanthine accumulated in XOR-R groups. PRPP was not detectable in inhibitor-treated groups (**a**, **b**; circled with an orange dashed line). ATP, ADP, AMP, IMP, and adenine levels were approximately 20–40% higher in XOR-R than in vehicle-treated rats in the reperfused state (B; circled with a black dashed line). Allopurinol treatment decreased succinyl AMP levels, whereas it increased orotic acid, orotidine 5′-P, and carbamoyl-Asp levels (**a**, **b**; circled with a red dashed line). PRPP, phosphoribosyl diphosphate; ATP, adenosine triphosphate; ADP, adenosine diphosphate; AMP, adenosine 5′-monophosphate; IMP, inosinic acid; GTP, guanosine triphosphate; GDP, guanosine 5′-diphosphate; GMP, guanosine 5′-monophosphate; CTP, cytidine triphosphate; CDP, cytidine 5′-diphosphate; CMP, cytidine 5′-monophosphate; UDP, uridine diphosphate; UMP, uridine 5′-monophosphate; succinyl AMP, adenylosuccinic acid; orotidine 5′-P, orotidine 5′-monophosphate; carbamoyl-Asp, N-carbamoylasparatic acid

A CE-TOFMS metabolomics analytical methods were also taken on kidney tissue lysates to observe overall metabolic changes on purine metabolism by XOR inhibitors, using the experimental set-up outlined in Table 1. Same as the results of HPLC analysis, there were 1.2- to 1.4-fold increase in ATP, ADP, AMP, IMP, and adenine in the inhibitor-administrated groups (Feb-R, Top-R, and Allo-R) compared to the Veh-R group in the reperfused state (metabolites circled with a black dashed line in Fig. 3b). Allopurinol treatment markedly decreased adenylosuccinic acid (succinyl AMP) levels in the resting and reperfused state, whereas orotic acid, orotidine 5′-monophosphate (orotidine 5′-P), and N-carbamoylaspartic acid (carbamoyl-Asp) levels were increased (metabolites circled with a red dashed line in Fig. 3a, b).

**Fig. 4:**
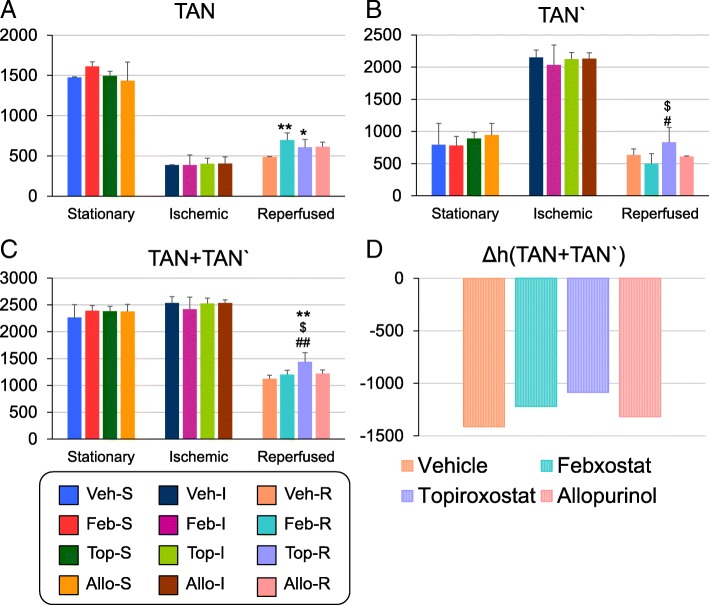
Quantification of TAN, TAN', and TAN + TAN' by CE-TOFMS. **a** Quantitative CE-TOFMS data for TAN coincided with the HPLC results. **b** TAN' was increased at ischemic state in contrast to the decrease in TAN, and decreased again after reperfusion. **c** The values of TAN+TAN’ did not significantly differ at stationary state among all groups and were well preserved at ischemic state; nevertheless, this parameter was decreased to as low as one half of that in the stationary state in the reperfused phase. **d** ΔhTAN + TAN' appeared to be lower in the febusostat and topiroxostat groups than in the vehicle and allopurinol groups. TAN = ATP + ADP + AMP), TAN = dATP + phosphoribosyl diphosphate (PRPP) + adenosine + adenine + inosine + IMP + hypoxanthine + xanthine + uric acid, ΔhTAN + TAN' was calculated as the TAN + TAN' of the reperfused state minus that of the ischemic state. Concentrations are expressed as nmol/g wet weight; the data are presented as the mean ± SEM (*n* = 5/6). *P < 0.05 and **P < 0.01 versus Veh; $*P* < 0.05 versus Feb; #P < 0.05 and ##*P* < 0.01 versus Allo; one-way ANOVA followed by Games–Howell’s test

The absolute concentrations of dATP, PRPP, adenosine, adenine, inosine, and IMP in addition to the compounds detected by HPLC analysis, were measured (Additional file 1: Table S2). All groups in the ischemic state showed an increase in IMP level and a decrease in dATP level, and a decrease in the adenine level after reperfusion. Notably, adenine was 1.28–1.71-fold more abundant in the XOR inhibitor-treated group than in the vehicle group. Here, we define TAN' as the sum of all detectable purine metabolites excluding ATP, ADP, and AMP, thus including dATP, PRPP, adenosine, adenine, inosine, IMP, hypoxanthine, xanthine, and uric acid (Fig. 4b). Then, TAN + TAN' represents the sum of major adenine compounds in a sample, which decreased to about half of its value in the stationary state after reperfusion (Fig. 4c). The Δh (TAN + TAN') value was defined the sum of reduction in purine nucleotide levels upon reperfusion, calculated as the TAN + TAN' in the reperfused state minus that in ischemic state. The Δh (TAN + TAN') values were smaller in the non-purine-analog XOR inhibitor-treated groups than in vehicle- and allopurinol-treated groups (Fig. 4d).

### CE-TOFMS metabolomics analysis reveals global metabolic alternations by I/R injury

**Fig. 5:**
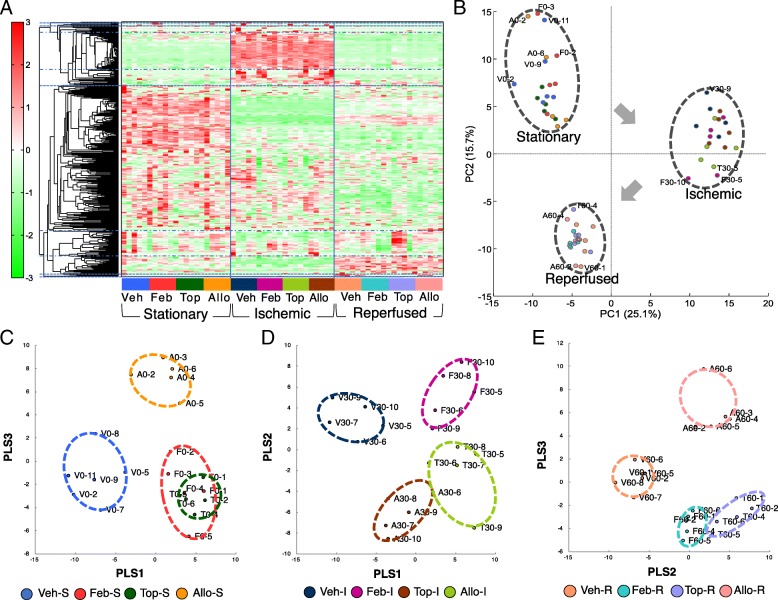
Heatmap, PCA and PLS-ROG analysis. **a** Heat map analysis of all samples showed that the kidney metabolic profile was altered mainly by I/R injury rather than XOR inhibitor interventions. **b** PCA results coincided with heat map data in that PC1 and PC2 clearly distinguished metabolic alterations by hemodynamic changes; each of the stationary, ischemic, and reperfused states formed a separate cluster. Partial least squares with rank order of groups (PLS-ROG) analysis of samples in the stationary, ischemic and reperfused states. **c** Based on PLS-ROG analysis in the stationary state, febuxostat-treated (Feb-S) and topiroxostat-treated (Top-S) samples show rather similar metabolic alterations but different from those in the two other groups. Vehicle-treated samples (Veh-S) had a metabolic profile distinguishable from those of XOR inhibitor-treated groups (Feb-S, Top-S, and Allo-S) by factor loadings for PLS1. Allopurinol-treated samples (Allo-S) were distinguishable from the other groups by factor loadings for PLS3. **d** In the ischemic state, vehicle-treated samples (Veh-I) had a metabolic profile distinguishable from those of XOR-inhibitor-treated groups (Feb-I, Top-I, and Allo-I) by factor loadings for PLS1. **e** In the reperfused state, vehicle-treated samples (Veh-R) had a metabolic profile distinguishable from those of XOR-inhibitor treated groups (Feb-R, Top-R, and Allo-R) by factor loadings for PLS2. Allopurinol-treated samples (Allo-R) were distinguishable from the other groups by factor loadings for PLS3

Global metabolomics survey was also taken by CE-TOFMS analysis to explore kidney metabolic alterations by XOR inhibition and/or I/R injury (Additional file 1: Figure S2). In total, 351 peaks were identified according to m/z and MT values. Of these, 180 peaks were detected in cationic mode and 171 in anionic mode. Heatmap analysis and PCA were conducted using all peaks to obtain a rough picture of the impacts of XOR inhibitor treatment and I/R injury on the metabolome (Fig. 5a, b). The results of heatmap and PCA analysis clearly distinguished the drastic metabolic changes by the I/R procedure, implicating that the impacts of drug interventions were less distinct than those of I/R injury itself (Fig. 5a, b). Representative contributing metabolites to such distinctions included triphosphate compounds in purine/pyrimidine metabolism pathways, nicotinamide adenine dinucleotide (NAD^+^), uridine diphosphate (UDP)-glucose, kynurenine, citrulline and amino acids such as ornithine, isoleucine, leucine, and tryptophan (Fig. 5b; for specific factor loadings see Additional file 1: Table S3 and S4).

Partial least squares with rank order of groups (PLS-ROG) and volcano plot analyses, which provide higher sensitivity in distinguishing differences between groups than PCA, were applied (Fig. 5c-e, Additional file 1: Figure S3–5). PLS-ROG analysis in the stationary state revealed that Veh-S and Allo-S were distinguishable from the non-purine-analog XOR inhibitor-treated groups; Feb-S and Top-S (Fig. 5c). The changes in levels of metabolites of purine metabolism pathways, namely the abundances of hypoxanthine, guanine, xanthine, and ATP, and reduced levels of uric acid and allantoin distinguished XOR inhibitor-treated groups well from Veh-S group (Fig. 5c, Additional file 1: Figure S3A-(i)). Meanwhile, the changes in levels of metabolites of pyrimidine pathways, namely increases in orotic acid, oxypurinol, xanthosine, and orotidine 5′-monophosphate distinguished the Veh-S and non-purine-analog XOR inhibitor-treated groups well from Allo-S group (Fig. 5c, Additional file 1: Figure S3A-(ii)).

As observed in the stationary state, PLS-ROG analysis distinguished vehicle groups (Veh-I and Veh-R) well from the allopurinol treated groups (Allo-I and Allo-R) and non-purine-analog XOR inhibitor-treated groups (Feb-I, Top-I, and Feb-S, Top-S) in the ischemic and reperfused phases (Fig. 5d, e and Additional file 1: Figure S4A-(i), 5A-(i)). The trends persisted under ischemic and reperfused conditions, in that the changes in levels of metabolites of pyrimidinemetabolism pathways were main factor loadings to distinguish vehicle and non-purine-analog XOR inhibitor-treated groups from allopurinol-treated groups (Fig. 5d, e and Additional file 1: Figure S4A-(ii), 5A-(iii)).

## Discussion

In this study, the dosage of three XOR inhibitors were designated to completely block XDH activities in renal tissues to contradict significant differences in their potencies against XOR; whose pIC50 values are 7.52 for febuxostat, 8.3 for topiroxostat and 6.0 for allopurinol, respectively. As a result, kidney XOR activity was suppressed to below the limit of detection in all inhibitor-treated groups, and blood and renal uric acid concentrations remained quite low and did not significantly differ among the inhibitor-treated groups. Therefore, it was demonstrated that each XOR inhibitor showed comparably potent inhibition of XDH in both kidneys and plasma, and alternations on metabolic profiles by administrating XOR inhibitors resulted probably not from the differences in XOR inhibition potencies.

Multiple reports support a relationship between organ disorder caused by I/R injury and reactive oxygen species (ROS) production (Tsuda et al. 2012; McCord 1985), and the major hypothesis for organ protection by XOR inhibitors is that the suppression of the conversion of XDH to XO under stressful conditions results in oxidative-stress suppression (Tsuda et al. 2012; Omori et al. 2012; McCord 1985). However, this does not fully explain the higher effectiveness of non-purine-analog XOR inhibitors than allopurinol (Sezai et al. 2015; Kim et al. 2017; Chou et al. 2017; Foody et al. 2017; Shafik 2013; Wang et al. 2015; Khan et al. 2017; Kato et al. 2016). Furthermore, no significant change in total XOR activity or elevation of XO-type activity was observed in vehicle-treated group, and western blotting yielded no evidence of production of XO-type proteins by limited proteolysis of XOR in I/R injury in accordance with previous reports (Okabe 1996), which is contradictory to oxidative-stress hypothesis (Tsuda et al. 2012; Omori et al. 2012; McCord 1985). Therefore, it is unlikely that the main locus of pathophysiology in I/R injury model was the conversion of XDH to XO triggered by physical stress nor the differences in their potencies of XOR inhibitors.

CE-TOFMS identified 351 metabolites, a number comparable to that in a previous renal tissue metabolome study (Wei et al. 2014), suggesting appropriate tissue handling and measurement. As ATP catabolism proceeds during tissue sampling and extraction, we assessed energy charge value to evaluate whether or not the sample processing was adequate as the values reflect the relative concentration of high-energy phosphates (Atkinson 1968). The levels were approximately 80% in the control stationary state, which is high when compared to previously reported levels (Khatib et al. 2001; Okabe 1996). Thus, sampling was considered to have been performed appropriately.

To summarize, the drastic metabolic changes by the I/R injury were much more significant than the impacts by administrating any XOR-inhibitors. The representative contributing factors for alternations of metabolites through I/R injury were continual decreases of high-energy purine compounds in purine/pyrimidine metabolism pathways, NAD^+^ and UDP-glucose, and consistent/transient increases of kynurenine, citrulline, amino acids and marked accumulation of hydrolysis products; such as lactate and β-hydroxybutyrate (Additional file 1: Figure S6A–C and Table S3 and S4), as reported previously (Weiner 1987). Those changes in the levels of such metabolites are considered result of anaerobic condition in renal tissues by I/R injury, as they play important roles in energy metabolism under static state.

The purine and pyrimidine metabolic pathways were most strongly affected by drug administration as proven by PLS-ROG and volcano analysis. Marked decreases in ATP and ADP and accumulation of hypoxanthine, xanthine, and uric acid during ischemia reflected a rapid loss of high-energy phosphates by anaerobic metabolism (purine degradation), in agreement with previous reports (Lasley et al. 1988; Stromski et al. 1986; Okabe 1996). Hypoxanthine and xanthine levels were higher, whereas uric acid and allantoin levels were lower in all XOR inhibitor-treated groups (with no difference among them) than in the vehicle group, and these are considered the results from inhibition of XDH activities by XOR inhibitors. The greater values of ATP, ADP and TAN in non-purine-analog XOR inhibitors were observed in the stationary state. High-energy phosphates markedly decreased during ischemia, with no significant differences between groups, while abundancy of such metabolites reappeared in the reperfused state. The less significant increases induced by non-purine-analog XOR inhibitors in the reperfused state than in the stationary state may have the following reasons: Firstly, reperfused samples are more likely to show greater variability than stationary samples as they pass through the I/R injury procedure, which can lead to individual differences and greater standard deviation (SD) value. Secondly, as the reperfused organs are about to resynthesize high-energy phosphates, they were probably in the earlier stage, judging from the low ATP recovery rates. Therefore, longer observation periods, i.e., 4 to 6 h. and/or 24 to 48 h. after I/R injury, and greater sample volumes per group may enhance pharmacological effects of XOR inhibitors and facilitate between-group differences. The barely comparable mRNA expressions of key enzymes for purine and pyrimidine metabolism between groups implied that these metabolic changes were not induced enzymatically.

**Fig. 6:**
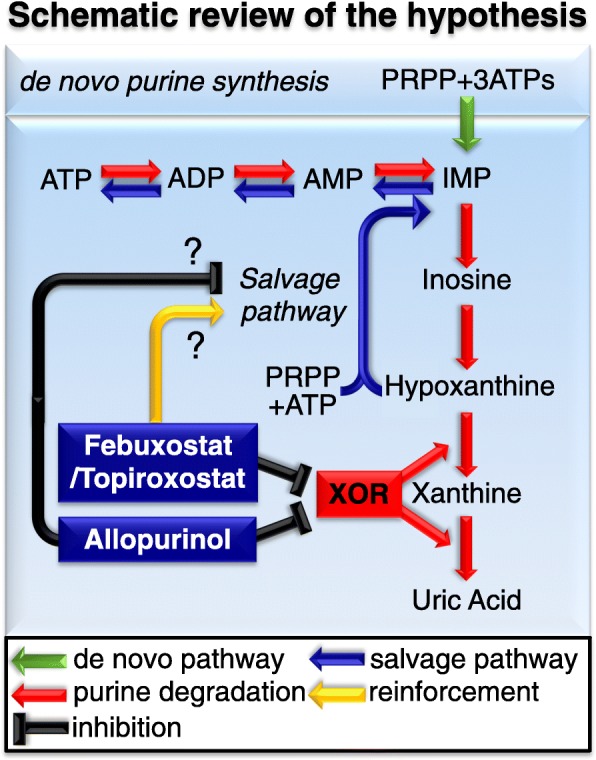
Schematic review of the hypothesis. When an organ is exposed to anaerobic condition, high-energy phosphates are degraded, and accumulation of xanthine and uric acid are usually greater than hypoxanthine in tissues through conversion of hypoxanthine to xanthine, and xanthine to uric acid by XDH activity. After reperfusion, these purine metabolites are washed out from tissues without XOR inhibition. Meanwhile, administration of non-purine-XOR inhibitors induce hypoxanthine accumulation in tissues by inhibiting XDH. After reperfusion, this excessive hypoxanthine is converted to IMP by hypoxanthine-guanine phosphoribosyltransferase (HGPRTase), a salvage pathway enzyme, which will be utilized for ATP resynthesis, and thus contribute to better energetic outcomes. As allopurinol itself act as a substrate for HGPRTase consuming PRPP, administration of allopurinol may inhibit ATP resynthesis via the salvage pathway. PRPP, phosphoribosyl diphosphate; ATP, adenosine triphosphate; ADP, adenosine diphosphate; AMP, adenosine 5′-monophosphate; IMP, inosine monophosphate; XOR, xanthine oxidoreductase

As the increases in levels of high-energy phosphates, such as ATP and TAN, have been previously reported to have organ-protective effects (Stromski et al. 1986; Stromski et al. 1988), our results may explain pathophysiology of organ-protective effects by XOR inhibitors, especially non-purine-analog XOR inhibitors. We propose the following hypothesis to explain the phenomenon described above (Fig. 6): When an organ is exposed to anaerobic condition, high-energy phosphates are degraded, and accumulation of xanthine and uric acid are usually greater than hypoxanthine in tissues through conversion of hypoxanthine to xanthine, and xanthine to uric acid by XDH activity. After reperfusion, these purine metabolites are washed out from tissues without XDH inhibition. Meanwhile, administration of XOR inhibitors induce hypoxanthine accumulation in tissues by inhibiting XDH. After reperfusion, this excessive hypoxanthine is converted to IMP by hypoxanthine-guanine phosphoribosyltransferase (HGPRTase), a salvage pathway enzyme, which will be utilized for ATP resynthesis, and thus contribute to better energetic outcomes. The smaller amount of Δh (TAN + TAN') value in the non-purine-analog XOR inhibitor-treated groups than in vehicle- and allopurinol-treated groups may represent these favorable effects by non-purine-analog XOR inhibitors, reflecting smaller amount of purine metabolites’ depletion through I/R injury (Fig. 4d). Also, as the presence of excess hypoxanthine in tissues is reported to preserve tissue injury during the brain I/R injury (Mink and Johnston 2007), an increase in the hypoxanthine concentration in renal tissue by XOR inhibitors is expected to have similar effects. The result of human HPRT enzyme kinetic data (Xu et al. 1997) implicates increase in IMP production in direct proportion to the 20 times increased hypoxanthine concentration, nevertheless confirming incorporation of excessive hypoxanthine for the resynthesis of high-energy phosphate compounds through the salvage pathway, for example, by using stable isotope tracing, would be necessary to strengthen the likelihood of the proposed hypothesis.

The reason why concentrations of ATP and TAN in the allopurinol-treated group were lower than those in the non-purine-analog XOR inhibitor groups throughout the I/R procedure may be because allopurinol affects other purine and pyrimidine metabolic enzymes (Okamoto et al. 2003; Takano et al. 2005; Becker et al. 2005). As allopurinol is structurally similar to hypoxanthine, it is metabolized extensively by XOR to oxypurinol, acting as a suicide substrate for XDH to generate oxypurinol; an isomer of xanthine (Additional file 1: Figure S7A-D) (Okamoto et al. 2003; Takano et al. 2005). Also, allopurinol can act as a substrate for HGPRTase (Nelson et al. 1973), yielding allopurinol-1-ribotide, which is decomposed to allopurinol-1-riboside and allopurinol, with partial consumption of PRPP, by cycling of the reactions. Moreover, allopurinol acts as a substrate for orotate phosphoribosyltransferase, which is converted to orotidine-5′-monophosphate with partial consumption of PRPP (Takano et al. 2005). Furthermore, allopurinol is converted to oxypurinol riboside, structurally similar to xanthosine (Additional file 1: Figure S7E, F), by reverse reaction of purine nucleoside phosphorylase (Krenitsky et al. 1967) with ribose-1-phosphate, a precursor of PRPP (Tozzi et al. 2006), thus reducing PRPP levels (Kato et al. 2016; Fox et al. 1970). Therefore, the increases in orotic acid, oxypurinol, xanthosine, and orotidine 5′-monophosphate as indicated by PLS-ROG analysis in allopurinol-treated group strongly indicates XOR-non-specific metabolic alternations and additional inhibitory effects on purine/pyrimidine enzymes by allopurinol. As PRPP and HGPRTase are required for the biosynthesis of IMP from hypoxanthine, administration of allopurinol itself may have suppressed the purine salvage pathway (Fig. 6). Although these unwelcoming affect by allopurinol on the purine salvage pathway needs further validation, this hypothesis is in good agreement with previous reports describing superiority of non-purine-analog XOR inhibitors over allopurinol in organ-protective effects (Sezai et al. 2015; Kim et al. 2017; Chou et al. 2017; Foody et al. 2017; Shafik 2013; Wang et al. 2015; Khan et al. 2017; Kato et al. 2016).

There are several limitations in our report. Firstly, as the clinical doses are 2–10% of doses administrated in our study, differences in the potency of XOR inhibition might influence the ability to suppress XOR activity more strongly at clinical practice. Secondly, purine metabolism differs between humans and rodents, in that XOR activity is much lower in humans than in rodents (Murase et al. 2016), while HGPRTase activity is higher in humans than in rodents (Tax and Veerkamp 1978). Finally, uric acid is further metabolized to allantoin by uricase, and excreted in the urine in rodents (Wu et al. 1992). In fact, allantoin production was suppressed by XOR inhibitor administration (Additional file 1: Figure S6F). Therefore, in-vitro experiments using human primary or iPS cells, and in-vivo studies using an animal model having physiological conditions and purine metabolism closer to those in humans, with lower XOR activity and uricase and higher HGPRTase activity, are desired.

## Conclusions

In conclusion, we evaluated XOR-inhibitor-induced metabolic alterations by CE-TOFMS. Importantly, the differences in purine/pyrimidine-pathway alterations between non-purine-analog XOR inhibitors and alopurinol were in accordance with our hypothesis that inhibition of XDH results in increases in the adenine nucleotide pool in renal tissues via the salvage pathway, whereas non-specific effects on enzymes by allopurinol may inhibit the alternative pathway. Thus, metabolically favorable changes induced by non-purine-analog XOR inhibitors may support organ-protective effects against metabolic, degenerative, and kidney diseases. Although further in-vivo and in-vitro experiments would be necessary to elucidate underlying mechanisms, we expect our hypothesis to be substantiated by further experiments, and non-purine-analog XOR inhibitors to be applied in the clinic as organ-protective agents in future.

## Additional file

Additional file 1 **Figure S1.** Gene expression of key enzymes for purine metabolism. **Figure S2.** Metabolic pathways of all detected metabolites. **Figure S3.** Top/Bottom Factor Loadings for PLS1/3 and volcano plot analysis under the stationary state. **Figure S4.** Top/Bottom Factor Loadings for PLS1/2 and volcano plot analysis under the ischemic state. **Figure S5.** Top/Bottom Factor Loadings for PLS2/3 and volcano plot analysis under the reperfused state. **Figure S6.** Relative peak areas of metabolites in kidney lysates as analyzed by CE-TOFMS. **Figure S7.** Structural similarities between allopurinol-associated metabolites and purine metabolites. **Table S1.** Concentrations of purine nucleotide as measured by HPLC system. **Table S2.** Quantitative evaluation of metabolites associated with purine nucleotide as measured by CETOF MS. **Table S3.** Top/Bottom Factor Loadings for PC1 in the stationary state. **Table S4.** Top/Bottom Factor Loadings for PC2 in the stationary state. (PDF 2410 kb)

## Data Availability

Please contact author for data requests.

## Abbreviations

ADP: Adenosine diphosphate
ALS: Amyotrophic lateral sclerosis
AMP: Adenosine 5′-monophosphate
ATP: Adenosine triphosphate
CDP: Cytidine 5′-diphosphate
CE-TOFMS: Capillary electrophoresis–time-of-flight mass spectrometry
CKD: Chronic kidney disease
CMP: Cytidine 5′-monophosphate
CTP: Cytidine triphosphate
eGFR: Glomerular filtration rate
GTP: Guanosine triphosphate
HGPRTase: Hypoxanthine-guanine phosphoribosyltransferase
HPLC: High-performance liquid chromatography
I/R: Ischemia-reperfusion
IMP: Inosine monophosphate
IMP: Intracellular inosine 5′-monophosphate
MT: Migration time
NAD^+^: Nicotinamide adenine dinucleotide
NADH: Nicotinamide adenine dinucleotide
PCA: Principal component analysis
PEP: Phosphoenolpyruvic acid
PRPP: Phosphoribosyl diphosphate
S7P: Sedoheptulose 7-phosphate
TAN: Total adenine nucleotide
UMP: Uridine 5′-monophosphate
UTP: Uridine triphosphate
XMP: Xanthosine 5′-phosphate
XO: Xanthine oxidase
XOR: Xanthine oxidoreductase
