# Supplementary material for: Metabolomics analysis elucidates unique influences on purine / pyrimidine metabolism by xanthine oxidoreductase inhibitors in a rat model of renal ischemia-reperfusion injury
Source: Mol Med. 2019 Aug 22;25:40. doi: 10.1186/s10020-019-0109-y (PMC6704627; doi:10.1186/s10020-019-0109-y)
Supplement: Supplementary file 1 — Figure S1. Gene expression of key enzymes for purine metabolism. Figure S2. Metabolic pathways of all detected metabolites. Figure S3. Top/Bottom Factor Loadings for PLS1/3 and volcano plot analysis under the stationary state. Figure S4. Top/Bottom Factor Loadings for PLS1/2 and volcano plot analysis under the ischemic state. Figure S5. Top/Bottom Factor Loadings for PLS2/3 and volcano plot analysis under the reperfused state. Figure S6. Relative peak areas of metabolites in kidney lysates as analyzed by CE-TOFMS. Figure S7. Structural similarities between allopurinol-associated metabolites and purine metabolites. Table S1. Concentrations of purine nucleotide as measured by HPLC system. Table S2. Quantitative evaluation of metabolites associated with purine nucleotide as measured by CETOF MS. Table S3. Top/Bottom Factor Loadings for PC1 in the stationary state. Table S4. Top/Bottom Factor Loadings for PC2 in the stationary state. (PDF 2410 kb) [file 10020_2019_109_MOESM1_ESM.pdf]

SUPPORTING INFORMATION

Metabolomics analysis elucidates unique influences on purine / pyrimidine metabolism by xanthine oxidoreductase inhibitors in a rat model of renal ischemia-reperfusion injury

Takashi Tani[1,2*], *Ken Okamoto*[2*], *Megumi Fujiwara*[2], *Akira Katayama*[2], *Shuichi Tsuruoka*[1]

[1]Department of Nephrology, Graduate School of Medicine, Nippon Medical School, 1-1-5 Sendagi, Bunkyo-ku, Tokyo, 113-8602 Japan

[2]Department of Metabolism and Nutrition, Graduate School of Medicine, Nippon Medical School, 1-1-5 Sendagi, Bunkyo-ku, Tokyo, 113-8602 Japan

*Correspondence to: Takashi Tani
Department of Nephrology, Graduate School of Medicine, Nippon Medical School, 1-1-5 Sendagi, Bunkyo-ku, Tokyo, 113-8602 Japan
Tel: +81-3-3822-2131
Fax: +81-3-5685-3054
E-mail: tani@nms.ac.jp

Overview

Supplementary Materials and Methods: Page 3-7

Additional File 1 –Figures: Page 8-14

Figure S1	Gene expression of key enzymes for purine metabolism	Page 8
Figure S2	Metabolic pathways of all detected metabolites	Page 9
Figure S3	Top/Bottom Factor Loadings for PLS1/3 and volcano plot analysis under the stationary state	Page 10
Figure S4	Top/Bottom Factor Loadings for PLS1/2 and volcano plot analysis under the ischemic state	Page 11
Figure S5	Top/Bottom Factor Loadings for PLS2/3 and volcano plot analysis under the reperfused state	Page 12
Figure S6	Relative peak areas of metabolites in kidney lysates as analyzed by CE-TOFMS	Page 13
Figure S7	Structural similarities between allopurinol-associated metabolites and purine metabolites	Page 14

Additional File 1 –Tables: Page 15-18

Table S1	Concentrations of purine nucleotide as measured by HPLC system	Page 15
Table S2	Quantitative evaluation of metabolites associated with purine nucleotide as measured by CE-TOF MS	Page 16
Table S3	Top/Bottom Factor Loadings for PC1 in the stationary state	Page 17
Table S4	Top/Bottom Factor Loadings for PC2 in the stationary state	Page 18

SUPPLEMENTARY MATERIALS AND METHODS

Drugs

Febuxostat (2-[3-cyano-4-isobutoxyphenyl]-4-methyl-1,3-thiazole-5-carboxylic acid; CAS: 144060-53-7) and allopurinol (4-hydroxypyrazolo[3,4-d]pyrimidine; CAS: 315-30-0) were purchased from Sigma-Aldrich (St. Louis, MO, USA), and topiroxostat (4-(5-pyridin-4-yl-1H-[1,2,4] triazol-3-yl)pyridine-2-carbonitrile) was kindly provided by Fuji Yakuhin (Saitama, Japan). Each XOR inhibitor (5 mg) was pulverized in a mortar and suspended in a small amount of 0.5% methylcellulose. Subsequently, 0.5% methylcellulose was added up to 10 ml.

Standards

For quantitative high-performance liquid chromatography (HPLC) and CE-TOFMS analyses, ATP disodium salt hydrate (CAS: 34369-07-8, F.W. 551.14, 27-1006-01; GE healthcare, Waukesha, WI, USA), ADP sodium salt (CAS: 20398-34-9, F.W. 427.20, A2754; Sigma-Aldrich), AMP disodium salt (CAS: 18422-05-4, F.W. 365.24, A2252; Sigma-Aldrich), hypoxanthine (CAS: 68-94-0, H9377; Sigma-Aldrich), xanthine (CAS: 69-89-6, 241-00013; Wako Pure Chemical Industries, Osaka, Japan), uric acid (CAS: 69-93-2, U2625; Sigma-Aldrich), oxypurinol (CAS: 2465-69-0, O6881; Sigma-Aldrich), and allopurinol (CAS: 315-30-0, A8003; Sigma-Aldrich) were used as standards.

Animals

Male Sprague–Dawley rats weighing 180–210 g (CLEA Japan Inc., Tokyo, Japan) were housed in standard cages with wood chip bedding at constant ambient temperature (21–22°C) and humidity (40–50%) with a 12-h light cycle. All animals had free access to tap water and the assigned diet. Prior to the start of the study, all mice were acclimatized for 7 days.

Measurement of purine nucleotide concentration by HPLC

Kidney extract (100 µl) was centrifugally concentrated and resuspended in 100 µL of buffer A (50 mM potassium phosphate buffer, pH 6.0). Samples were filtered through a 0.22-µm Millipore filter (Merck KGaA, Darmstadt, Germany), and 10 µl was injected into a HPLC system (ÄKTApurifier UPC 10; GE Healthcare UK/Amersham, Little Chalfont, Buckinghamshire, UK). A reverse-phase column (Supelcosil LC-18-T, 250 × 4.6 mm, 5 µm;

Sigma-Aldrich, Bellefonte, PA, USA) protected with a guard column (Supelguard LC-18-T, 20 × 4.0 mm; Sigma-Aldrich) was used. For peak analysis and quantification, UNICORN version 5.0 (GE Healthcare) was used. Chromatograms were obtained at wavelengths of 250, 260, and 295 nm. Buffer A and buffer B (50 mM potassium phosphate buffer and 10% methanol) were prepared in deionized water and filtered through a 0.22-μm filter before use. The flow rate was 1.3 ml/min, and the gradient profile was as follows: 9 min in 100% buffer A, 15 min to 25% buffer B, 17.5 min to 90% buffer B and maintained until 23.5 min, and 35 min to 0% buffer B. The run was maintained at 100% buffer B for an additional 3 min before it was completed and the gradient was returned to 100% buffer A. Concentrations were calculated by comparing sample peak areas with standard peak areas. Standard curves were drawn using 3.125 µM to 200 µM standard solutions prepared in buffer A. All concentrations are expressed as nmol/g wet weight.

Measurement of metabolites

Samples (600 □l each) were added to 240 µL of Milli-Q water containing internal standards (Solution ID: H3304-1002; Human Metabolome Technologies, Tsuruoka, Japan) on ice to inactivate enzymes. The mixture (800 □l) was filtered through a Millipore 5-kDa cutoff filter at 9,100 ×*g* and 4°C for 60 min to remove proteins. The filtrate was centrifugally concentrated and resuspended in 50 µL of Milli-Q water for CE-MS. Metabolome measurements were performed at Human Metabolome Technologies.

CE-TOFMS was performed on an Agilent CE Capillary Electrophoresis System equipped with an Agilent 6210 TOF mass spectrometer, Agilent 1100 isocratic HPLC pump, Agilent G1603A CE-MS adapter kit, and Agilent G1607A CE-ESI-MS sprayer kit (Agilent Technologies, Waldbronn, Germany). The systems were controlled by Agilent i ChemStation software version B.03.01 for CE (Agilent Technologies, Waldbronn, Germany). The metabolites were analyzed on a fused silica capillary (50 µm *i.d.* × 80 cm total length), with commercial electrophoresis buffer (Solution ID: H3301-1001 for cation analysis and H3302-1021 for anion analysis; Human Metabolome Technologies) as the electrolyte. The sample was injected at a pressure of 50 mbar for 10 s (~10 nL) in cation analysis and 25 s (~25 nL) in anion analysis. The scan range was *m/z* 50–1,000. Other conditions were as described previously (1-3).

Data processing and analysis

Peaks were extracted using MasterHands software (Keio University, Tsuruoka, Japan) to obtain peak information, including *m/z*, migration time for CE-TOFMS measurement (MT), and peak area (4, 5). Signal peaks corresponding to isotopomers, adduct ions, and other product ions of known metabolites were excluded, and the remaining peaks were annotated to putative metabolites from the HMT metabolite database based on their migration time (MT) and *m/z* values determined by TOFMS. The tolerance range for peak annotation was set at ±0.5 min for MT and ±10 ppm for *m/z*. In addition, the MT was normalized to those of internal standards, and peaks were aligned according to the *m/z* values and normalized MT values. Finally, peak areas were normalized to those of the internal standards, methionine sulfone and D-camphor-10-sulfonic acid, for cations and anions, respectively. The resultant relative area values were further normalized to sample amount. Annotation tables were produced by CE-TOFMS measurement of standard compounds and were aligned with the datasets according to similar *m/z* values and normalized MT values. The relative peak areas were calculated using the following equation:

$$\text{Relative peak area} = \frac{\text{Metabolite peak area}}{\text{Internal standard peak area} \times \text{Sample amount}}$$

The peak detection limit was determined based on the signal-noise ratio: S/N = 3.

Western blotting

We followed the method used in our previous reports, with minor modifications (6, 7). Frozen tissue samples were homogenized in ice-cold 50 mM potassium phosphate buffer (pH 7.8) containing 0.4 mM EDTA and complete protease inhibitor cocktail (Roche Diagnostics, Indianapolis, IN, USA). Tissue lysates were centrifuged (10 min, 4°C, 20,670 ×*g*), and the supernatants were used for western blotting and XOR activity assay. The total protein concentration was determined using a DC Protein Assay kit (Bio-Rad Laboratories, Hercules, CA, USA). After boiling at 95°C for 5 min, 10 µg of each protein sample was resolved by sodium dodecyl sulfate polyacrylamide gel electrophoresis (Mini-PROTEAN TGX 4–15%, Bio-Rad Laboratories), and bands were electrophoretically transferred to a 0.2-µm-thick polyvinylidene fluoride membrane (Trans-Blot Turbo; Bio-Rad Laboratories). The membrane was blocked in 5% skim milk TBS-T (50 mM Tris-buffered saline with 0.05% Tween-20) for 1 h. After washing with TBS-T, polyclonal rabbit antibody (6) was added for 1 h (1:1,000). The membrane was washed and incubated with horseradish peroxidase-conjugated polyclonal goat anti-rabbit

immunoglobulin antibody (1:10,000; Dako Denmark A/S, Glostrup, Denmark) for 1 h. Signals were visualized with an ECL kit (GE Healthcare, Fairfield, CT, USA), and analyzed with the ChemiDoc XRS Plus System with Image Lab SVP Software (Bio-Rad Laboratories).

Quantitative reverse-transcription (qRT-)PCR

RNA was extracted from frozen tissue samples using an RNeasy Mini Kit (Qiagen GmbH, Hilden, Germany) according to the manufacturer's protocol. The RNA concentration was determined on a NanoDrop 2000 (Thermo Scientific, Delaware, ME, USA). Complementary DNA was generated with oligo-dT primers using the ReverTra qPCR RT Kit (Toyobo Co., Osaka, Japan) according to the manufacturer's protocol. qPCR amplification was carried out using the TaqMan Fast Advanced Master Mix (Life Technologies, Carlsbad, CA, USA) and TaqMan gene expression assay probes in reaction mixtures of 20 □L. Probes for 18S RNA (Rn01428913_gH), xanthine dehydrogenase (Rn00567654_m1), adenine phosphoribosyl transferase (Rn01432775_m1), HGPRT 1 (Rn01527840_m1), and kidney injury molecule 1 (Rn00597703_m1) were purchased from Life Technologies. The TaqMan probes have a FAM fluorochrome reporter tag at the 5′-end and an MGB quencher at the 3′-end. Amplification was performed in 96-well optical plates on an ABI 7500 Fast Real-Time PCR System (Life Technologies), with one cycle at 95°C for 20 s, followed by 50 cycles at 95°C for 3 s and 60°C for 30 s. Each sample was analyzed in triplicate. Products were analyzed using the manufacturer's software (SDS 1.1). Gene expression was normalized to levels of 18S rRNA as an internal control and expressed as fold increases using the ΔΔCt method.

1. Soga T, Heiger DN. Amino acid analysis by capillary electrophoresis electrospray ionization mass spectrometry. Analytical chemistry. 2000;72(6):1236-41.

2. Soga T, Ueno Y, Naraoka H, Ohashi Y, Tomita M, Nishioka T. Simultaneous determination of anionic intermediates for Bacillus subtilis metabolic pathways by capillary electrophoresis electrospray ionization mass spectrometry. Analytical chemistry. 2002;74(10):2233-9.

3. Soga T, Ohashi Y, Ueno Y, Naraoka H, Tomita M, Nishioka T. Quantitative metabolome analysis using capillary electrophoresis mass spectrometry. Journal of proteome research. 2003;2(5):488-94.

4. Sugimoto M, Wong DT, Hirayama A, Soga T, Tomita M. Capillary electrophoresis mass spectrometry-based saliva metabolomics identified oral, breast and pancreatic cancer-specific

profiles. Metabolomics : Official journal of the Metabolomic Society. 2010;6(1):78-95.
5. Subramanian P, Oh BJ, Mani V, Lee JK, Lee CM, Sim JS, et al. Differential Metabolic Profiles during the Developmental Stages of Plant-Parasitic Nematode Meloidogyne incognita. International journal of molecular sciences. 2017;18(7).
6. Ikegami T, Nishino T. The presence of desulfo xanthine dehydrogenase in purified and crude enzyme preparations from rat liver. Archives of biochemistry and biophysics. 1986;247(2):254-60.
7. H.Okabe. The role of xanthine dehydrogenase (xanthine oxidase) in ischemia-reperfusion injury in rat kidney. Jpn J Nephrol. 1996;38:577-84.

Figure S1 Gene expression of key enzymes for purine metabolism.

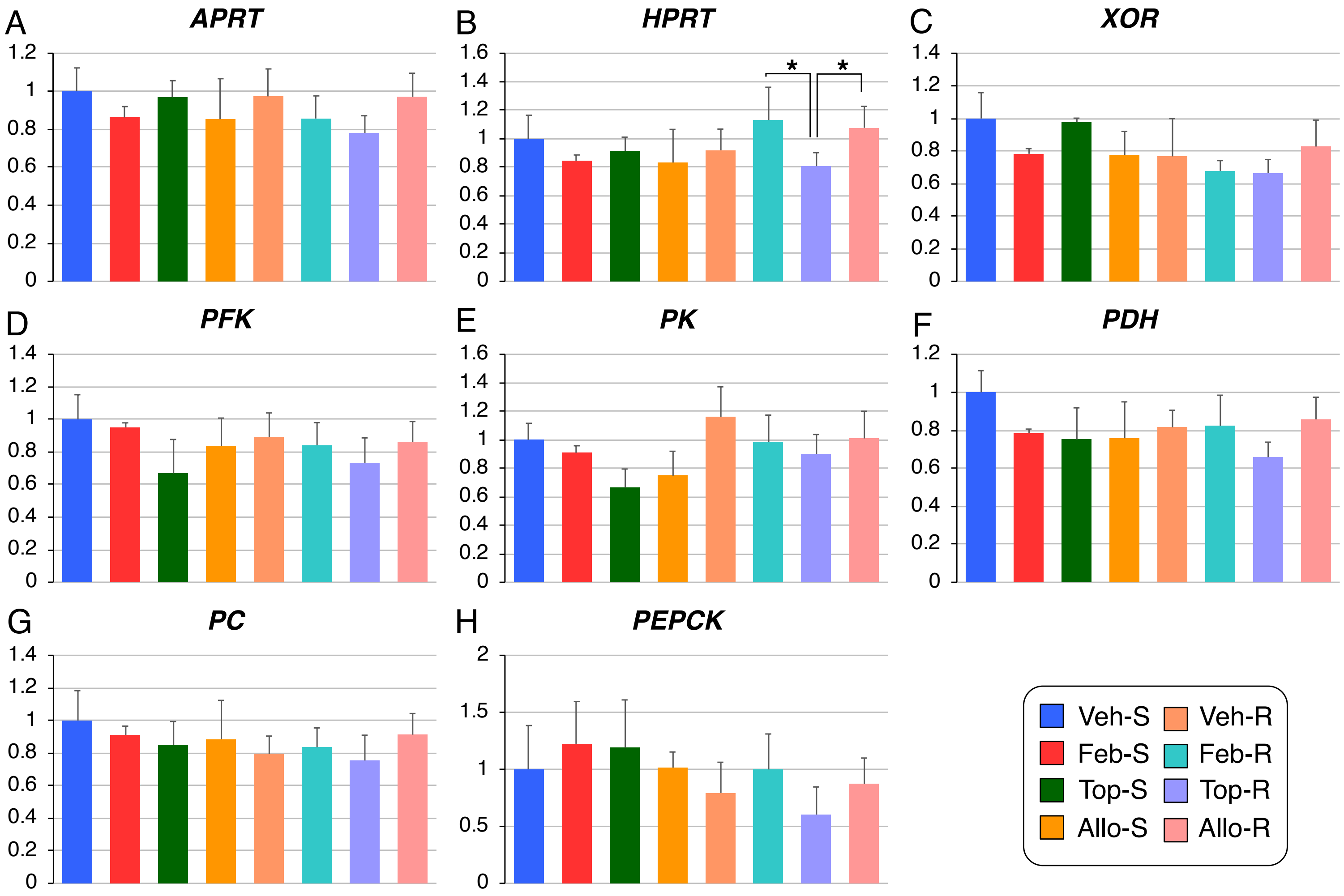

We evaluated the mRNA expression of key enzymes in the stationary and reperfused phases: **(A)** mRNA expression levels of adenine phosphoribosyltransferase (*APRT*), **(B)** hypoxanthine phosphoribosyltransferase (*HPRT*), **(C)** xanthine oxidoreductase (*XOR*), **(D)** phosphofructokinase (*PFK*), **(E)** pyruvate kinase (*PK*), **(F)** pyruvate dehydrogenase kinase (*PDH*), **(G)** pyruvate carboxylase (*PC*)**,** and **(H)** phosphoenolpyruvate carboxykinase (*PEPCK*) were determined by qPCR in triplicate, normalized to 18S rRNA levels, and expressed relative to expression levels in the Veh-S group, set as 1. **(B)** Gene expression of *HPRT* was decreased in the Top-R group as compared to the Feb-R and Allo-R groups. No other significant differences within groups in the stationary and reperfused phases were observed. Data represent the mean ± SEM (n = 5–6). *$P < 0.05$ **$P < 0.01$; non-repeated measures ANOVA.

Figure S2 Metabolic pathways of all detected metabolites.

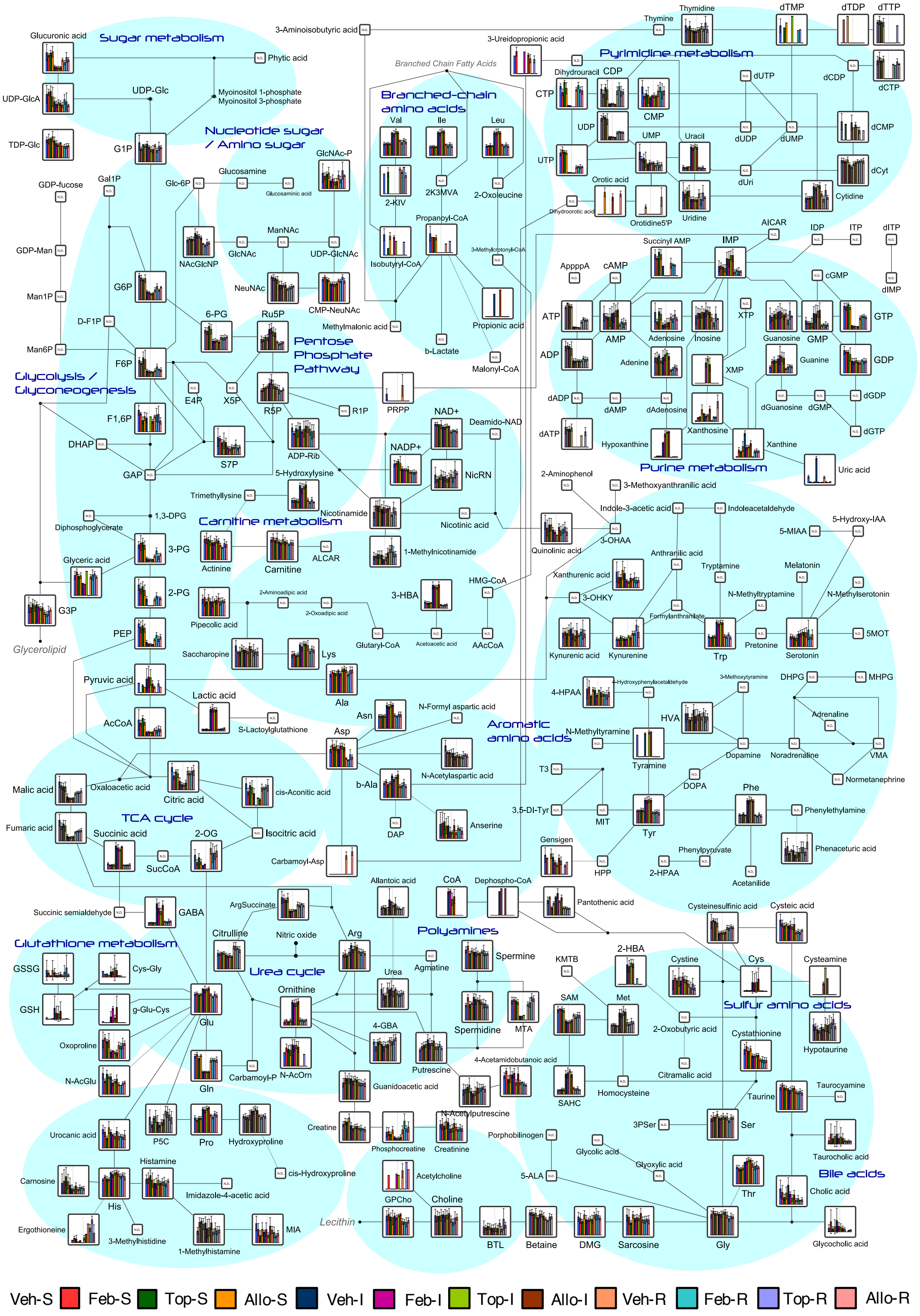

Relative peak area values of detected metabolites were plotted on metabolic pathway maps. The metabolic pathways involved were associated mainly with glycolysis/gluconeogenesis, the pentose phosphate pathway, the tricarboxylic acid cycle, the urea cycle, purine and pyrimidine metabolism, nicotinate/nicotinamide metabolism, and amino acid metabolism

Figure S3 Top/Bottom Factor Loadings for PLS1/3 and Volcano plot analysis under stationary state.

A (i)

	Top Factor Loadings for PLS1 under stationary state			
	Top 10 metabolites		Bottom 10 metabolites	
1	Hypoxanthine	0.895	Uric acid	-0.949
2	Guanine	0.824	Allantoin	-0.797
3	1-Methylhistamine	0.725	Trimethylamine N-oxide	-0.667
4	Xanthine	0.615	N-Acetylaspartic acid	-0.659
5	Putrescine	0.573	Succinic acid	-0.611
6	N-Acetylornithine	0.556	Citric acid	-0.602
7	Pyridoxamine 5'-phosphate	0.556	Betonicine	-0.584
8	Ethanolamine phosphate	0.551	5-Methyl-2'-deoxycytidine	-0.563
9	ATP	0.537	Saccharopine	-0.561
10	3-Guanidinopropionic acid	0.517	CMP	-0.549

ATP, adenosine triphosphate; CMP, cytidine 5'-monophosphate.

(ii)

	Top Factor Loadings for PLS3 under stationary state			
	Top 10 metabolites		Bottom 10 metabolites	
1	Orotic acid	0.972	N-Acetylglycine	-0.522
2	Oxypurinol	0.849	threo-β-Methylaspartic acid	-0.521
3	Xanthosine	0.804	Lactic acid	-0.519
4	Orotidine 5'-monophosphate	0.709	UDP-N-acetylgalactosamine	-0.442
5	Suberic acid	0.679	ADP-glucose	-0.438
6	Triethanolamine	0.576	Phenylalanine	-0.437
7	Xanthine	0.570	Adenine	-0.434
8	Creatine	0.529	Methionine	-0.418
9	Urocanic acid	0.473	N-Acetyl-β-alanine	-0.377
10	XC0040	0.453	XA0017	-0.375

ADP, adenosine diphosphate; UDP, uridine diphosphate.

B

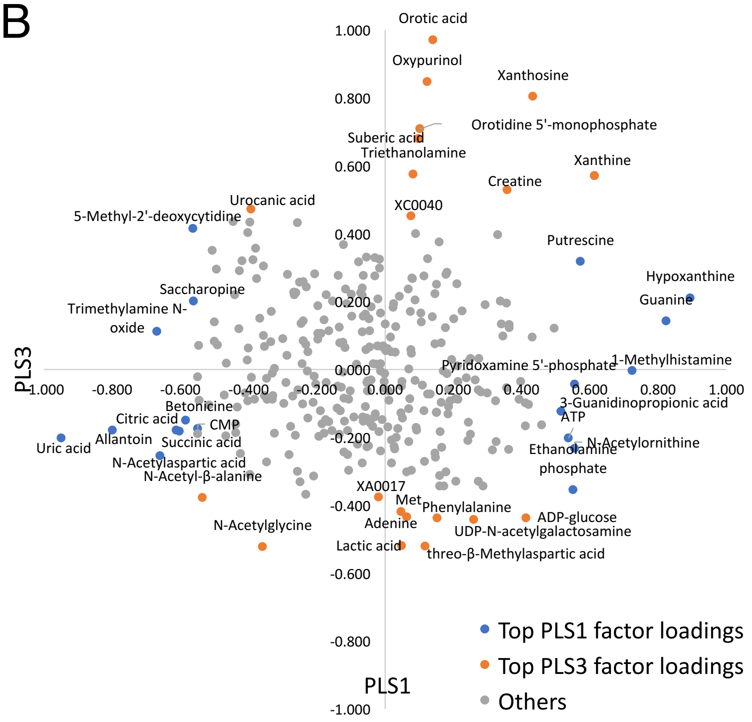

C D

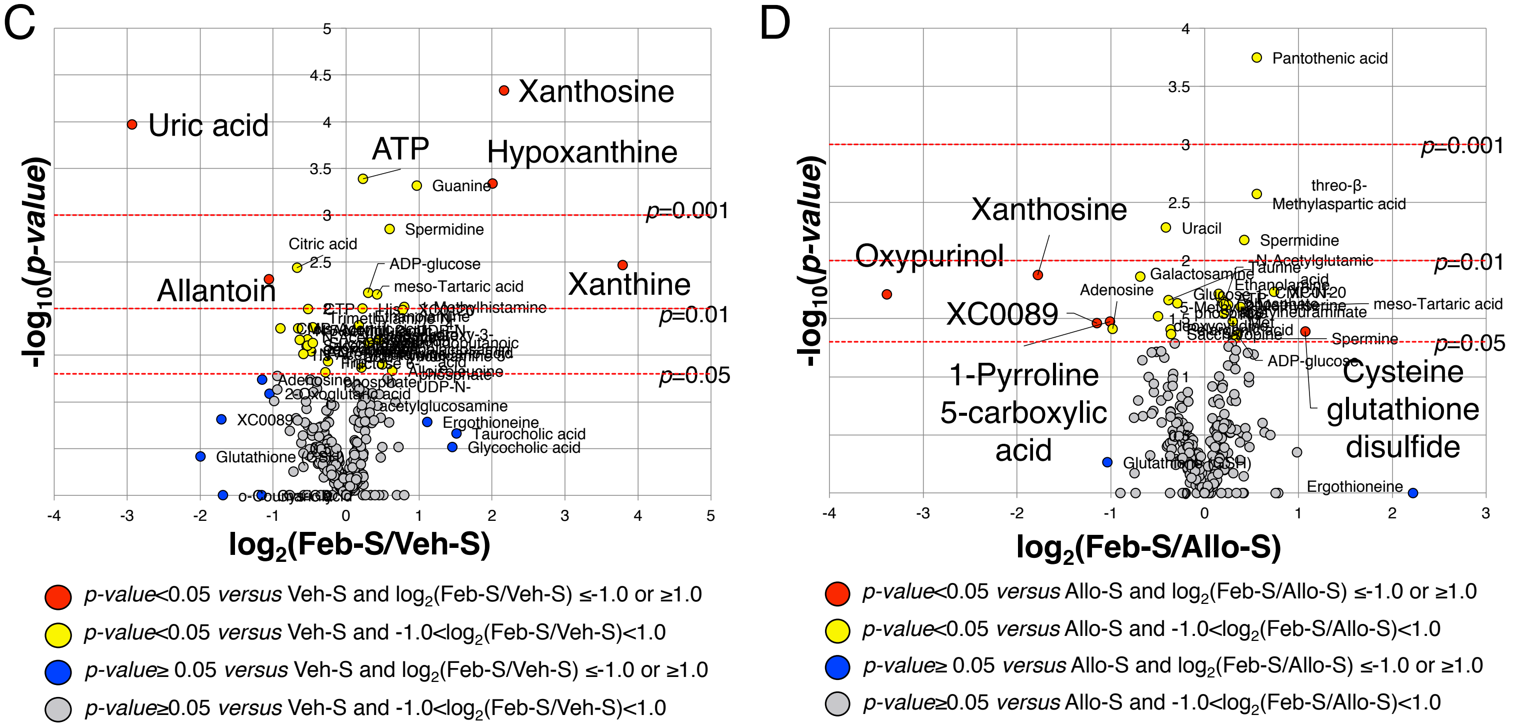

(A) Top/Bottom 10 factor loadings for PLS1 and PLS3 under stationary state by partial least squares with rank order of groups (PLS-ROG) analysis. **(B)** The loadings bi-plot of the top ranked loadings with the PLS-ORG scores. **(C)** In the volcano plot, more than two-fold increases/decreases in hypoxanthine, xanthosine, xanthine, allantoin, and uric acid levels in the Feb-S group compared to the Veh-S group were observed. **(D)** The Feb-S group showed increases and decreases in cysteine glutathione disulfide, 1-pyrroline 5-carboxylic acid, XC0089, xanthosine, and oxypurinol by more than two-fold as compared to the Allo-S group. XC0089 was an undetermined peak, but was predicted to be 2-hydroxyfelbamate.

Figure S4 Top/Bottom Factor Loadings for PLS1/2 and Volcano plot analysis under ischemic state.

A (i)

Top Factor Loadings for PLS1 under ischemic state

	Top 10 metabolites		Bottom 10 metabolites	
1	Hypoxanthine	0.759	Xanthine	-0.905
2	XMP	0.609	Uric acid	-0.855
3	CMP	0.492	Allantoin	-0.845
4	Betaine	0.469	Xanthurenic acid	-0.735
5	2-Methylserine	0.455	Glutaric acid	-0.705
6	Asparagine	0.430	5-Methylcytosine	-0.698
7	Isobutyryl CoA_divalent	0.420	Cytosine	-0.687
8	Threonine	0.418	Cholic acid	-0.667
9	Dyphylline	0.385	Sedoheptulose 7-phosphate	-0.656
10	2,6-Diaminopimelic acid	0.360	3-Indoxylsulfuric acid	-0.652

CMP, cytidine 5'-monophosphate; XMP, xanthosine 5'-phosphate.

(ii)

Top Factor Loadings for PLS2 under ischemic state

	Top 10 metabolites		Bottom 10 metabolites	
1	Indole-3-acetic acid	0.589	Oxypurinol	-0.782
2	CTP	0.505	2'-Deoxycytidine	-0.698
3	Glucose 6-phosphate	0.502	Xanthosine	-0.696
4	XA0004	0.500	Orotic acid	-0.679
5	UTP	0.495	Alanine	-0.656
6	Pelargonic acid	0.484	N-Acetylputrescine	-0.518
7	5'-Deoxy-5'-methylthioadenosine	0.483	XC0132	-0.513
8	Glucose 1-phosphate	0.477	Glycerophosphocholine	-0.495
9	Pantothenic acid	0.464	Hypoxanthine	-0.491
10	Benzoylformic acid	0.454	β-Alanine	-0.484

CTP, cytidine triphosphate; UTP, uridine triphosphate.

B

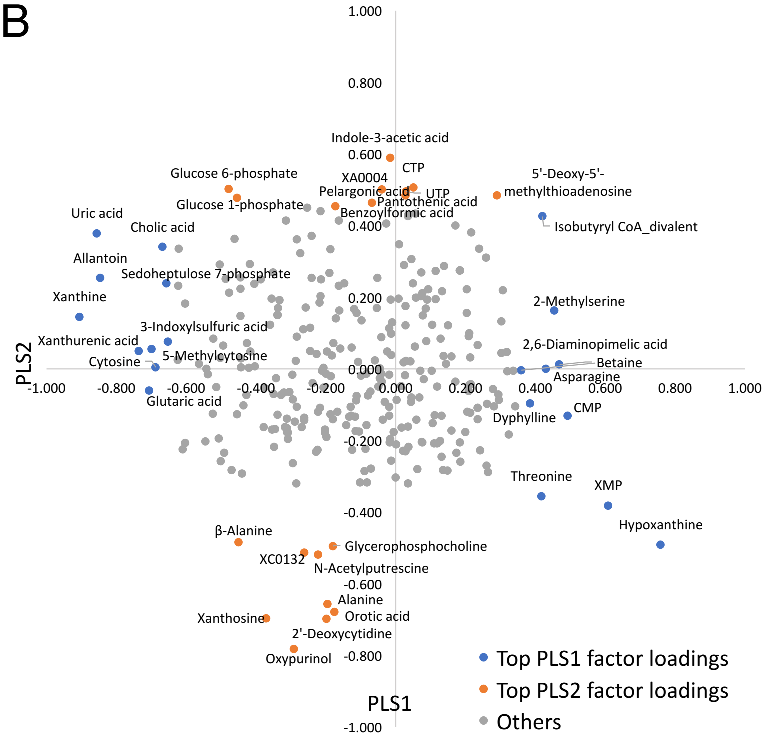

C

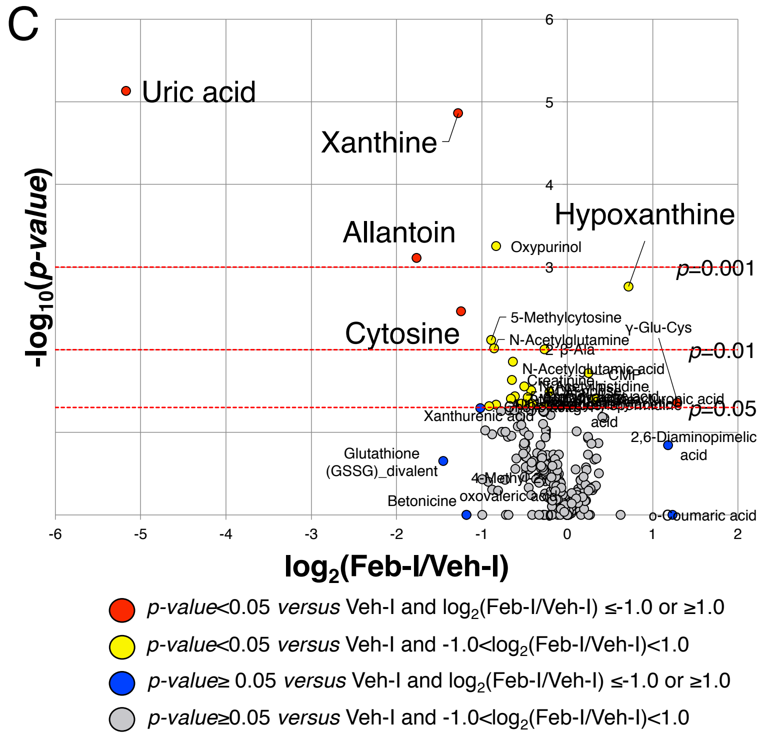

D

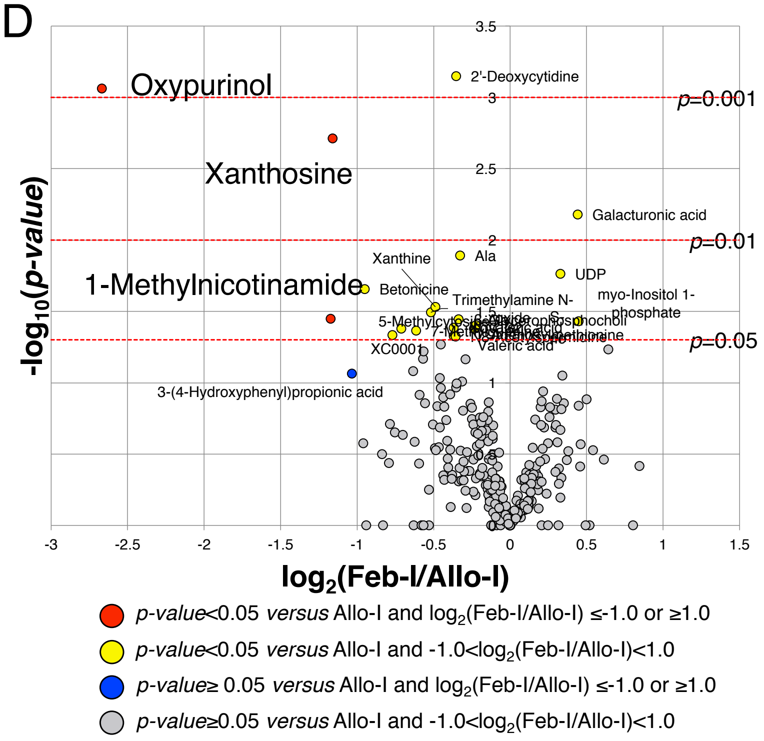

(A) Top/Bottom 10 factor loadings for PLS1 and PLS2 under ischemic state by partial least squares with rank order of groups (PLS-ROG) analysis. **(B)** The loadings bi-plot of the top ranked loadings with the PLS-ORG scores. **(C)** In the volcano plot, more than two-fold decreases in cytosine, xanthine, allantoin, and uric acid levels in the Feb-I group as compared to the Veh-I group were observed. **(D)** The Feb-I showed more than two-fold decreases in xanthosine, oxypurinol, and 1-methylnicotinamide levels as compared to the Allo-I group.

Figure S5 Top/Bottom Factor Loadings for PLS2/3 and Volcano plot analysis under reperfused state.

A (i)

Top Factor Loadings for PLS2 under reperfused state

	Top 10 metabolites		Bottom 10 metabolites	
1	Xanthine	0.881	Uric acid	-0.925
2	Hypoxanthine	0.876	Allantoin	-0.892
3	Guanine	0.681	PRPP	-0.597
4	Mucic acid	0.668	o-Hydroxyhippuric acid	-0.547
5	N-Acetylneuraminic acid	0.666	1-Methyl-4-imidazoleacetic acid	-0.508
6	Adenine	0.647	2-Hydroxyisobutyric acid	-0.480
7	5'-Deoxy-5'-methylthioadenosine	0.644	N-Carbamoylaspartic acid	-0.443
8	N-Glycolylneuraminic acid	0.600	4-Acetamidobutanoic acid	-0.428
9	Sulfotyrosine	0.570	Methylguanidine	-0.419
10	XA0033	0.562	Guanidinosuccinic acid	-0.415

PRPP, phosphoenolpyruvic acid.

(ii)

Top Factor Loadings for PLS3 under reperfused state

	Top 10 metabolites		Bottom 10 metabolites	
1	Oxypurinol	0.858	1-Methylnicotinamide	-0.425
2	Orotidine 5'-monophosphate	0.791	4-Acetamidobutanoic acid	-0.424
3	Xanthosine	0.753	UDP-glucuronic acid	-0.405
4	N-Carbamoylaspartic acid	0.572	CTP	-0.401
5	Orotic acid	0.522	Glyceric acid	-0.399
6	Phenaceturic acid	0.488	Glutaric acid	-0.397
7	3-Indoxylsulfuric acid	0.420	2-Hydroxyisobutyric acid	-0.397
8	XC0132	0.418	Tryptophan	-0.380
9	2-Aminoisobutyric acid	0.410	Adenylosuccinic acid	-0.379
10	Lactic acid	0.386	β-Alanine	-0.368

CTP, cytidine triphosphate; UDP, uridine diphosphate.

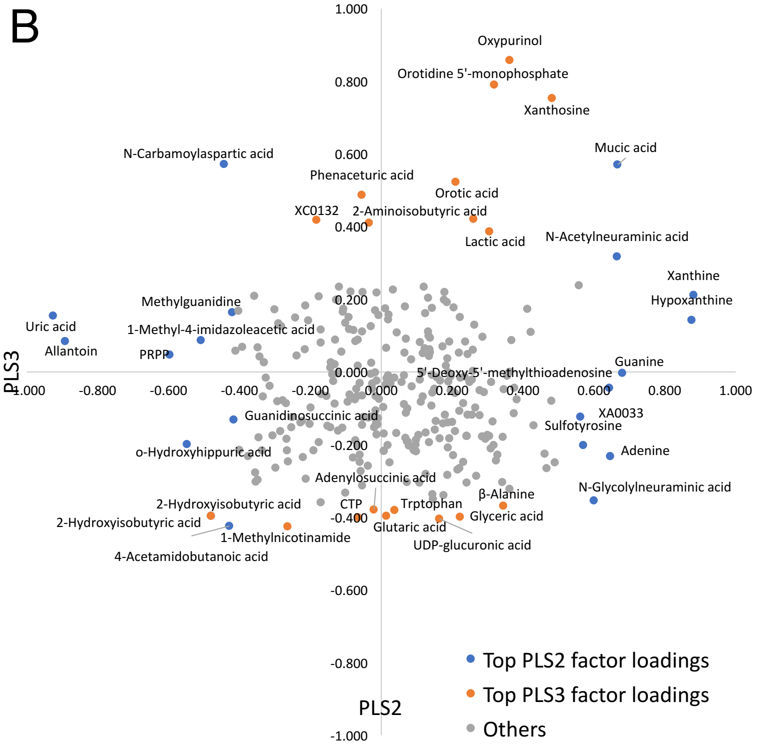

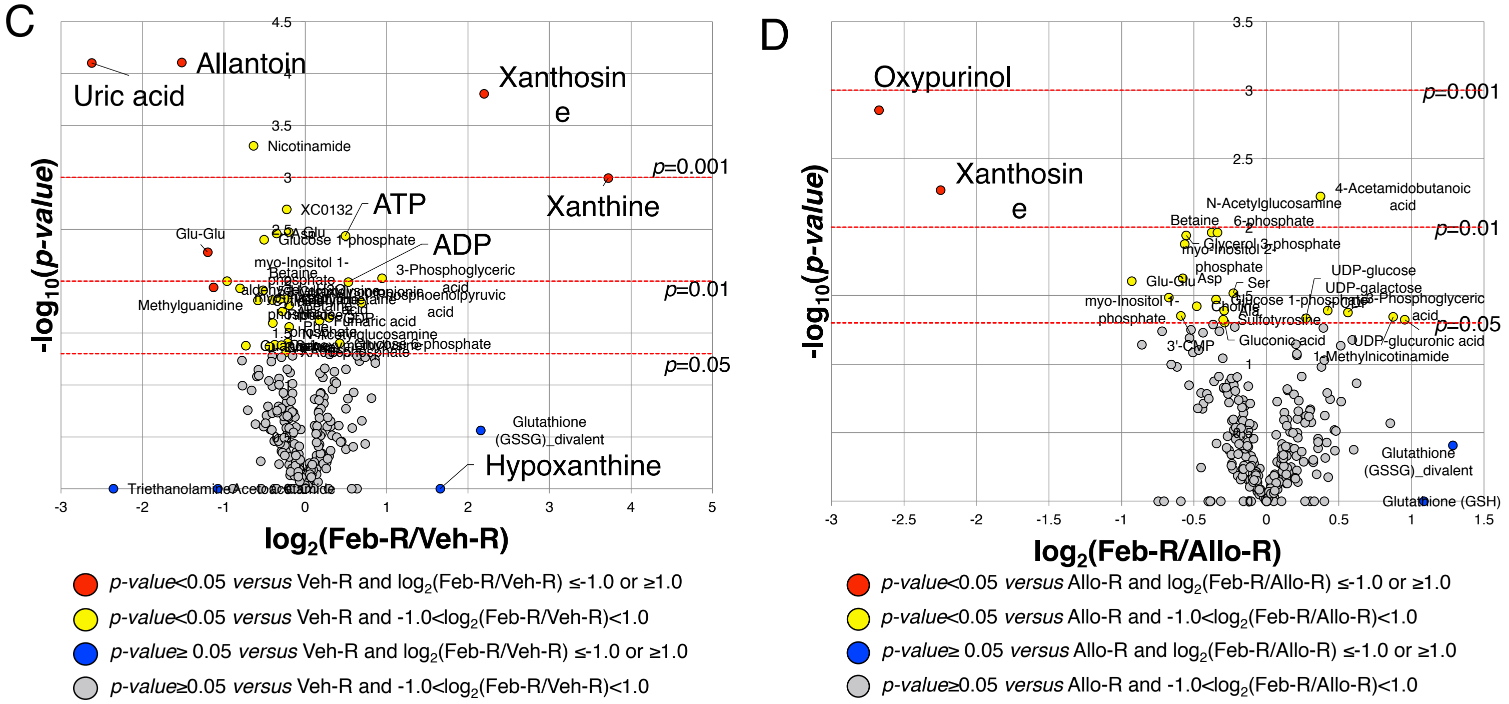

(A) Top/Bottom 10 factor loadings for PLS2 and PLS3 under reperfused state by partial least squares with rank order of groups (PLS-ROG) analysis. **(B)** The loadings bi-plot of the top ranked loadings with the PLS-ORG scores. **(C)** In the volcano plot, more than two-fold increases/decreases in xanthosine, xanthine, allantoin, and uric acid levels in the Feb-R group compared to the Veh-R group were observed. **(D)** The Feb-R group showed more than two-fold decreases in xanthosine and oxypurinol as compared to Allo-R.

Figure S6 Relative peak areas of metabolites in kidney lysates as analyzed by CE-TOFMS.

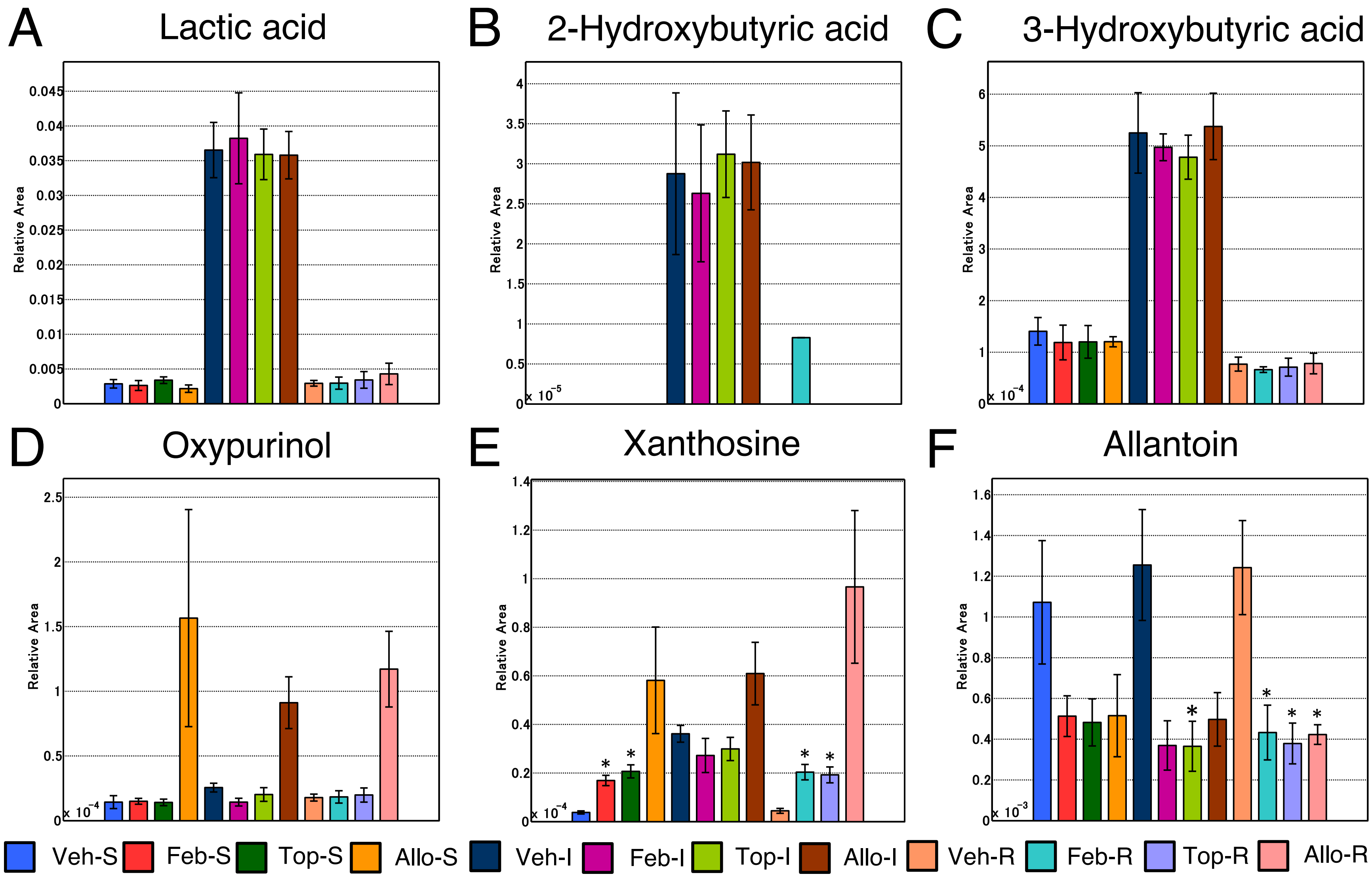

(A,B,C) Lactic acid, 2-hydroxybutyric acid, 3-hydroxybutyric acid levels were greatly increased during ischemia with no significant differences between groups. **(D)** Oxypurinol tended to be increased only in allopurinol-administrated groups. **(E)** Levels of xanthosine tended to be increased by XOR inhibitor administration, especially allopurinol, at all time points: This may be due to the co-existence of oxypurinol-riboside in the renal tissue, which is an isomer of xanthosine and the metabolite of allopurinol. **(F)** Levels of allantoin showed tendency of decreases by XOR inhibitor administration at all time points. q-value was quantified by Benjamini-Hochberg procedure and was used for the statistical cut-off of significance: *$q < 0.05$ *versus* Veh group.

Figure S7 Structural similarities between allopurinol-associated metabolites and purine metabolites.

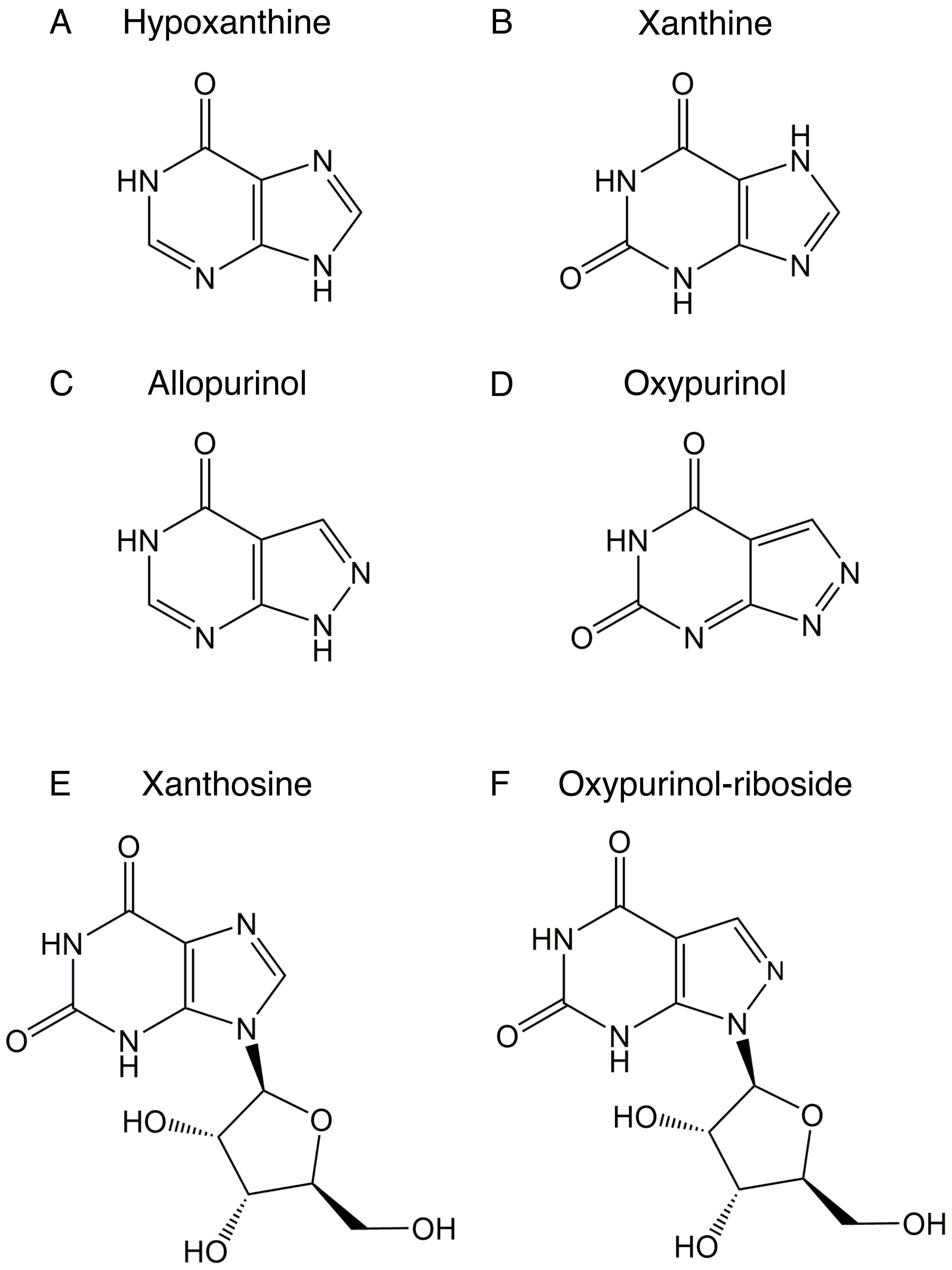

Structural similarities between hypoxanthine, xanthine, allopurinol and oxypurinol **(A,B,C,D),** and xanthosine and allopurinol riboside **(E,F)** are shown.

Table S1 Concentrations of purine nucleotide as measured by HPLC system

	Stationary				Ischemic				Reperfused			
	Veh-S	Feb-S	Top-S	Allo-S	Veh-I	Feb-I	Top-I	Allo-I	Veh-R	Feb-R	Top-R	Allo-R
Energy Charge(%)	73.9 ±5.5	74.0 ±2.1	76.4 ±2.2	75.1 ±4.6	38.5 ±5.5	38.0 ±7.4	34.4 ±3.8	33.0 ±3.5	68.7 ±5.2	69.2 ±1.4	70.3 ±6.0	68.7 ±2.2
TAN	1293 ±158	1503 ±161 ** ##	1485 ±43 * ##	1187 ±53	349 ±74	341 ±111	349 ±62	357 ±84	455 ±68	650 ±128 *	599 ±138	551 ±41
ATP	744 ±61	889 ±115 ** ##	908 ±30 ** ##	711 ±39	70.4 ±10.1	64.7 ±6.9	63.8 ±8.7	56.8 ±4.6	238 ±38	341 ±68 *	316 ±68	284 ±21
ADP	410 ±102	448 ±31	451 ±33 #	357 ±39	121 ±28	114 ±19	109 ±36	116 ±31	148 ±32	218 ±47	199 ±56	188 ±18
AMP	140 ±59	166 ±36	126 ±26	119 ±50	157 ±49	162 ±94	176 ±37	184 ±54	69.4 ±18.0	91.5 ±16.4	82.8 ±42.6	79.2 ±12.2
Hypoxanthine	N.D	N.D	12.5 ±15.3 ** #	N.D	620 ±23	1025 ±101 **	1060 ±52 **	999 ±55 **	N.D	8.7 ±10.7	9.8 ±12.0	5.7 ±11.4
Xanthine	N.D	73.8 ±50.0 **	68.0 ±19.5 ** #	122.8 ±45.5 **	466 ±9	195 ±52 **	201 ±28 **	240 ±32 **	N.D	92.7 ±18.5 **	111 ±27 **	89.5 ±21.6 **
Uric acid	83.5 ±31.2	5.58 ±4.2 **	0.73 ±0.9 **	3.85 ±1.3 **	363 ±25	11.0 ±3.3 **	3.8 ±1.3 **	10.5 ±1.5 **	87.9 ±12.7	13.5 ±4.9 **	3.7 ±0.8 **	13.7 ±2.2 **

Energy Charge and total adenine nucleotide were calculated by the following formula; energy charge =ATP+0.5ADP/ATP+ADP+AMP, TAN=ATP+ADP+AMP. Concentrations are expressed as nmol/g wet weight; the data are represented as the mean ± SEM (n=5~6); N.D: not detected. *, *p*<0.05 and **, *p*<0.01 *versus* Vehicle group; #, *p*<0.05 and ##, *p*<0.01 *versus* Allopurinol group; all *p*-values are evaluated by non-repeated measures ANOVA.

Table S2 Concentrations of purine nucleotide as measured by CE-TOF MS.

	Stationary				Ischemic				Reperfused			
	Veh-S	Feb-S	Top-S	Allo-S	Veh-I	Feb-I	Top-I	Allo-I	Veh-R	Feb-R	Top-R	Allo-R
Energy Charge(%)	75.2 ±5.4	78.8 ±1.9	78.2 ±2.6	78.6 ±5.7	40.0 ±7.1	38.5 ±6.7	35.5 ±5.5	33.8 ±4.7	76.6 ±4.6	75.3 ±2.5	76.2 ±6.8	74.7 ±4.0
TAN	1476 ±222	1612 ±105	1494 ±134	1433 ±291	387 ±124	385 ±160	403 ±90	407 ±97	487 ±86	699 ±109 **	607 ±162 *	613 ±88
TAN\`	792 ±332	783 ±140	891 ±96	945 ±181	2147 ±118	2033 ±309	2124 ±103	2132 ±91	635 ±96	501 ±157	833 ±229 $#	608 ±13
TAN+TAN\`	2268 ±235	2395 ±96	2384 ±88	2378 ±132	2535 ±120	2418 ±231	2527 ±100	2538 ±55	1122 ±67	1200 ±85	1440 ±167 ** $ ##	1221 ±66
ATP	908 ±55	1067 ±22 ***	972 ±64	948 ±75	89 ±18.2	85 ±17.8	78 ±9.1	73 ±4.0	310 ±49	435 ±54 **	385 ±67	377 ±38 *
ADP	402 ±89	407 ±54	392 ±38	358 ±124	132 ±33	126 ±43	131 ±21	128 ±21	127 ±20	182 ±30 *	154 ±47	163 ±27 *
AMP	166 ±78	138 ±29	129 ±32	127 ±93	166 ±73	174 ±99	195 ±60	205 ±72	50.4 ±17.5	81.2 ±24.8	67.6 ±48.4	73.4 ±24
dATP	1.41 ±0.5	1.27 ±0.1	1.27 ±0.2	1.37 ±0.3	N.D	N.D	N.D	N.D	0.80 N.A	N.D	1.09 ±0.3	N.D
PRPP	2.30 ±1.8	N.D	N.D	N.D	N.D	N.D	N.D	N.D	5.19 ±3.6	N.D	N.D	N.D
Adenosine	215 ±118	96.4 ±32.2	222 ±40	190 ±71	56.3 ±24.0	65.6 ±40.9	84.3 ±53.8	88.8 ±49.9	156 ±38	133 ±44	185 ±51	133 ±20
Adenine	3.73 ±0.9	3.72 ±1.17	4.17 ±0.3	3.07 ±0.5	3.83 ±1.4	4.36 ±1.2	4.73 ±0.5	4.00 ±0.6	1.09 ±0.2	1.39 ±0.3	1.86 ±0.4 **	1.55 ±0.5
Inosine	434 ±280	508 ±161	503 ±140	541 ±184	530 ±113	611 ±223	589 ±117	538 ±158	369 ±105	215 ±122	484 ±223	329 ±22
IMP	41.4 ±24.4	43.6 ±13.7	38.7 ±9.4	32.3 ±16.6	76.3 ±27.5	73.1 ±34.6	90.0 ±19.9	90.1 ±27.9	17.0 ±9.2	24.4 ±5.5	19.7 ±16.5	18.5 ±4.0
Hypoxanthine	12.7 ±4.7	51.1 ±10.4 ***	52.8 ±9.9 ***	51.3 ±9.3 ***	652 ±37	1073 ±138 **	1151 ±59 ***	1160 ±34 ***	6.8 N.A	21.3 ±6.1	26.8 ±6.3	22.7 ±7.3
Xanthine	N.D	73.8 ±50.0	68.0 ±19.5	123 ±45.5	466 ±8.9	195 ±53	201 ±28	240 ±32	N.D	92.7 ±18.5	111 ±27.0	89.5 ±21.6
Uric Acid	84 ±31	5.6 ±4.2	0.7 ±0.9	3.9 ±1.3	363 ±24.7	11.0 ±3.3	3.8 ±1.3	10.5 ±1.5	87.9 ±12.7	13.5 ±4.9	3.7 ±0.8	13.7 ±2.2

Energy charge, total adenine nucleotide and TAN\` were calculated by the following formula; energy charge =ATP+0.5ADP/ATP+ADP+AMP, TAN=ATP+ADP+AMP and TAN\`= dATP+phosphoribosyl diphosphate(PRPP)+adenosine+adenine+inosine+inosine monophosphate(IMP)+hypoxanthine+xanthine+uric acid. Concentrations are expressed as nmol/g wet weight; the data are represented as the mean ± SEM (n=5~6); N.D: not detected. *, P<0.05, **, P<0.01 and ***, P<0.001 *versus* Vehicle group; $, P<0.05 *versus* Febuxostat group; #, P<0.05 and ##, P<0.01 *versus* Allopurinol group; all P-values are evaluated by non-repeated measures ANOVA.

	Table S3. Top/Bottom Factor Loadings for PC1 (Figure 5B)			
	Top 20 metabolites		Bottom 20 metabolites	
1	Ornithine	0.104	L-Glutamine	-0.102
2	L-Isoleucine	0.103	CTP	-0.100
3	Uracil	0.103	5'-Deoxy-5'-methylthioadenosine	-0.099
4	O-Succinylhomoserine	0.102	Mucic acid	-0.097
5	Lactic acid	0.102	GTP	-0.096
6	L-Leucine	0.102	4-Methyl-2-oxovaleric acid	-0.094
7	L-Tryptophan	0.102	S-Adenosylmethionine	-0.093
8	L-Phenylalanine	0.102	CDP	-0.092
9	Succinic acid	0.101	ATP	-0.089
10	S-Adenosylhomocysteine	0.101	Lauric acid	-0.088
11	N-Acetylgalactosamine	0.101	Citric acid	-0.088
12	Isobutyric acid	0.101	Glyceric acid	-0.088
13	Hypoxanthine	0.101	Argininosuccinic acid	-0.088
14	L-Valine	0.101	Fumaric acid	-0.088
15	L-Asparagine	0.100	Phosphoenolpyruvic acid	-0.087
16	2-Hydroxybutyric acid	0.100	Malic acid	-0.086
17	3-Hydroxybutyric acid	0.100	Glucuronic acid	-0.086
18	5-Hydroxylysine	0.099	Glucose 6-phosphate	-0.086
19	Thiamine	0.098	UTP	-0.085
20	L-Methionine	0.098	Cysteinesulfinic acid	-0.085

ATP, adenosine triphosphate; CDP, cytidine 5'-diphosphate; CTP, cytidine triphosphate; GTP, guanosine triphosphate; UTP, uridine triphosphate.

Table S4. Top/Bottom Factor Loadings for PC2 (Figure 5B)

	Top 20 metabolites		Bottom 20 metabolites	
1	NAD^+	0.120	Kynurenine	-0.088
2	XA0033	0.117	Citrulline	-0.080
3	UDP-glucose	0.115	2-Oxoglutaric acid	-0.073
4	UDP-N-acetylgalactosamine	0.113	N-Acetylalanine	-0.072
5	Ethanolamine phosphate	0.112	1-Methyladenosine	-0.071
6	N-Acetylneuraminic acid	0.112	Pyridoxal	-0.067
7	Cystathionine	0.111	N-Carbamoylaspartic acid	-0.065
8	$NADP^+$	0.110	Dyphylline	-0.064
9	N-Glycolylneuraminic acid	0.106	N1-Methyl-4-pyridone-5-carboxamide	-0.063
10	O-Acetylhomoserine	0.106	Phosphocreatine	-0.061
11	L-Aspartic acid	0.105	XC0126	-0.056
12	Daminozide	0.104	4-Aminohippuric acid	-0.056
13	3-Hydroxy-3-methylglutaric acid	0.102	1-Methylnicotinamide	-0.053
14	Cysteic acid	0.101	Lauric acid	-0.052
15	Isoglutamic acid	0.098	1-Pyrroline-5-carboxylic acid	-0.050
16	Creatine	0.098	N6-Methyllysine	-0.049
17	7-Methylguanine	0.098	Ergothioneine	-0.046
18	Gulonic acid	0.097	myo-Inositol 2-phosphate	-0.045
19	Sulfotyrosine	0.096	Nicotinamide	-0.042
20	4-Acetamidobutanoic acid	0.096	2-Oxoisovaleric acid	-0.041

UDP, uridine diphosphate. XA0033 and XC0126 are undetermined peaks, but candidate metabolic molecules are predicted as follows. XA0033, cytidine; XC0126, N-acetylneuraminic acid.
